# Structure of the Large Extracellular Loop of FtsX and Its Interaction with the Essential Peptidoglycan Hydrolase PcsB in Streptococcus pneumoniae

**DOI:** 10.1128/mBio.02622-18

**Published:** 2019-01-29

**Authors:** Britta E. Rued, Martín Alcorlo, Katherine A. Edmonds, Siseth Martínez-Caballero, Daniel Straume, Yue Fu, Kevin E. Bruce, Hongwei Wu, Leiv S. Håvarstein, Juan A. Hermoso, Malcolm E. Winkler, David P. Giedroc

**Affiliations:** a Department of Biology, Indiana University Bloomington, Bloomington, Indiana, USA; b Department of Chemistry, Indiana University Bloomington, Bloomington, Indiana, USA; c Department of Crystallography and Structural Biology, Instituto Química-Física Rocasolano, Consejo Superior de Investigaciones Científicas, Madrid, Spain; d Department of Chemistry, Biotechnology and Food Science, Norwegian University of Life Sciences, Ås, Norway; University of Mississippi Medical Center; Newcastle University; University of Nottingham

**Keywords:** NMR structure, *Streptococcus pneumoniae*, cell division, peptidoglycan hydrolases, protein-protein interactions

## Abstract

Streptococcus pneumoniae is a leading killer of infants and immunocompromised adults and has become increasingly resistant to major antibiotics. Therefore, the development of new antibiotic strategies is desperately needed. Targeting bacterial cell division is one such strategy, specifically by targeting proteins that are essential for the synthesis and breakdown of peptidoglycan. One complex important to this process is FtsEX. FtsEX comprises a cell division-regulating integral membrane protein (FtsX) and a cytoplasmic ATPase (FtsE) that resembles an ATP-binding cassette (ABC) transporter. Here, we present nuclear magnetic resonance (NMR) solution structural and crystallographic models of the large extracellular domain of FtsX, denoted extracellular loop 1 (ECL1). The structure of ECL1 reveals an upper extended β-hairpin and a lower α-helical lobe, each extending from a mixed α-β core. The helical lobe mediates a physical interaction with the peptidoglycan hydrolase PcsB via the coiled-coil domain of PcsB (PscB_CC_). Characterization of S. pneumoniae strain D39-derived strains harboring mutations in the α-helical lobe shows that this subdomain is essential for cell viability and required for proper cell division of S. pneumoniae.

## Abstract (precis)

FtsX is a ubiquitous bacterial integral membrane protein involved in cell division that regulates the activity of peptidoglycan (PG) hydrolases. FtsX is representative of a large group of ABC3 superfamily proteins that function as “mechanotransmitters,” proteins that relay signals from the inside to the outside of the cell. Here, we present a structural characterization of the large extracellular loop, ECL1, of FtsX from the opportunistic human pathogen S. pneumoniae. We show the molecular nature of the direct interaction between the peptidoglycan hydrolase PcsB and FtsX and demonstrate that this interaction is essential for cell viability. As such, FtsX represents an attractive, conserved target for the development of new classes of antibiotics.

## INTRODUCTION

Streptococcus pneumoniae is a Gram-positive, opportunistic respiratory pathogen (1–3) that has acquired antibiotic resistance worldwide (4–6). This ovococcal bacterium relies on highly conserved cell wall machinery to divide and grow (7, 8). The cell wall is primarily composed of peptidoglycan (PG), a macromolecule composed of repeating subunits of *N*-acetylglucosamine and *N*-acetylmuramic acid linked by PG peptide side chains (9, 10). Regulation of the synthesis and remodeling of PG is essential for bacterial growth and viability, due to the turgor pressure bacterial cells must withstand (10–12). One vital process for the synthesis of PG is the controlled insertion of new strands of PG. This process requires timed cleavage of the old PG matrix to allow incorporation of new nascent strands (13). PG hydrolases are the primary enzymes that carry out PG cleavage and remodeling (14, 15). Thus, regulation of these hydrolases and activation at specific times during the cell cycle is required for proper cell growth. Specific protein complexes are utilized by bacterial cells to regulate these enzymes. This work focuses on understanding the structure and function of one of these protein complexes.

From Mycobacterium tuberculosis to Caulobacter crescentus, the ATP-binding cassette (ABC) transporter-like protein complex FtsEX acts as a key regulator of PG hydrolysis and divisome assembly (16–19). The proposed mechanism of FtsEX activation of PG hydrolases is as follows. FtsE, upon sensing an unknown signal from inside the cell, hydrolyzes ATP to ADP. Hydrolysis causes a conformational change that is transmitted through the membrane via FtsX, an integral membrane protein with two extracellular loops (ECLs), denoted the large (ECL1) and small (ECL2) loops. These extracellular loops interact with either cell wall hydrolases or effector proteins, which results in activation of PG hydrolysis via an unknown mechanism (16, 18, 20–25). In Escherichia coli, it has been demonstrated that FtsX interacts with the effector protein EnvC to activate the PG amidases AmiA and AmiB (24, 25). In addition, FtsX interacts with other division proteins, such as FtsA, where it regulates the polymerization of FtsA and recruitment of downstream division proteins (26). In other organisms, including Bacillus subtilis and M. tuberculosis, FtsEX also activates PG hydrolases (16, 23). Interestingly, FtsEX is nonessential in rod-shaped bacteria like E. coli and B. subtilis (23, 24, 26–28). However, in S. pneumoniae, FtsEX is absolutely essential (21) and depletion of FtsEX results in cell rounding and cessation of growth (20, 21).

In the case of S. pneumoniae, genetic experiments suggest that both outward-facing domains of FtsX, ECL1 and ECL2, interact with the essential PG hydrolase PcsB via its long coiled-coil (CC) domain (20, 21). However, there is little direct biochemical evidence for this interaction. ECL1 and ECL2 are thought to allosterically activate the catalytic activity of the cysteine, histidine-dependent amidohydrolase/peptidase (CHAP) domain of PcsB (20). The crystal structure of full-length PcsB, including the CC domain, an alanine-rich linker region, and the CHAP domain, provides insight into the mechanism of how this may occur (22). While the PcsB structure implies that FtsEX activates PcsB by displacing the catalytic domain from the CC domain, the exact nature of the FtsX-PcsB interaction remains unknown.

In order to understand how FtsX activates PcsB, we determined the structure of the large extracellular loop of FtsX (FtsX_ECL1_) by both multidimensional nuclear magnetic resonance (NMR) spectroscopy and X-ray crystallography. FtsX_ECL1_ harbors a conserved mixed α-β core and a lower α-helical lobe extending from the core, identified previously in M. tuberculosis FtsX (16), and S. pneumoniae FtsX_ECL1_ has a unique extended β-hairpin. The N-terminal β1 and C-terminal β6 strands are adjacent in the core and connect ECL1 to the transmembrane 1 (TM1) and TM2 helices, respectively, in the membrane. PscB_CC_-mediated chemical shift perturbations of spectra obtained by ^1^H-^15^N heteronuclear single quantum coherence (HSQC) spectroscopy of FtsX_ECL1_ reveal that the helical lobe consisting of the α2 helix and the α2-β5 linker (residues 107 to 134) of FtsX_ECL1_ interacts with PscB_CC_. To determine if this interaction is required for FtsX function in bacterial cells, we constructed a merodiploid strain that allows for conditional expression of mutant *ftsX*. We demonstrate that specific amino acid substitutions in the FtsX-PcsB interface are lethal or cause pronounced morphological defects despite the fact that these FtsX_ECL1_ mutant proteins are expressed at nearly wild-type levels. These findings support the model that a direct physical interaction between FtsX and PcsB is required for activation of PcsB PG hydrolytic activity.

## RESULTS

### The three-dimensional structure of FtsX_ECL1_.

**FIG 1:**
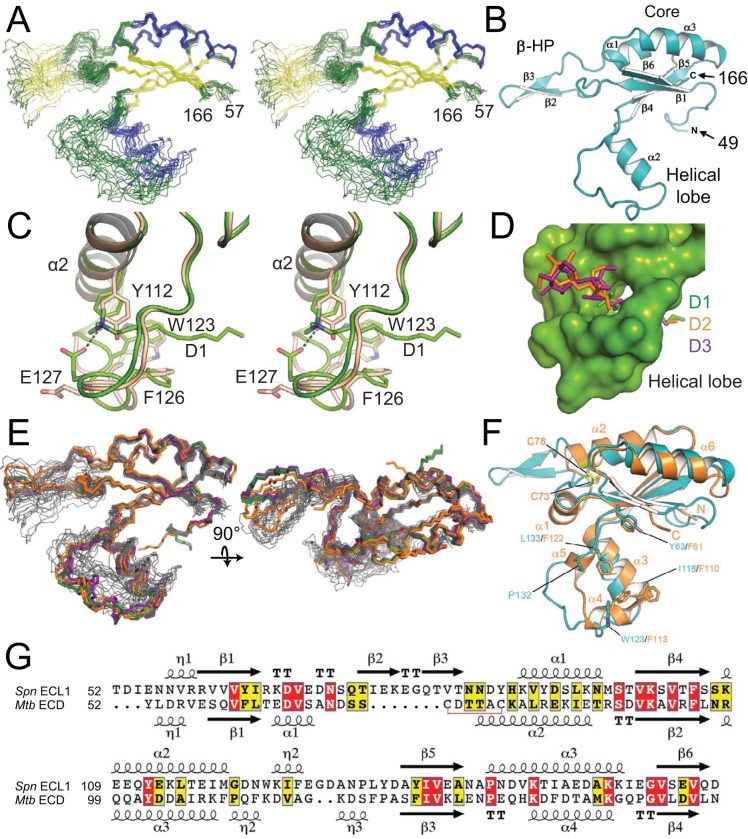
The structure of FtsX_ECL1_ from Streptococcus pneumoniae. (A) Stereoview of the 20 conformers of the FtsX_ECL1_ NMR structure as backbone traces, with helices shown in blue and β-strands shown in yellow. N and C termini of the domain are indicated by the residue numbers 57 and 166. (B) Cartoon representation of the FtsX_ECL1_ structure obtained by X-ray crystallography (chain B in FtsX_ECL1_-D1) in which the different secondary structure elements have been numbered and labeled. N and C termini of the domain are indicated by the residue numbers 49 and 166. β-HP, β-hairpin. (C) Stereoview showing changes in helical lobe upon interaction with dodecyltrimethylammonium chloride. Apo form (chain B) is colored in pale brown, and holo form (chain A) is colored in green. Relevant residues affected by the presence of the detergent are depicted as sticks. The polar interaction is represented by a dashed line. (D) Surface representation of the FtsX_ECL1_ crystal structure in which the three different detergent molecules are superimposed and shown in sticks, as follows: dodecyltrimethylammonium chloride, *n*-undecyl-β-d-maltoside, and *n*-decyl-β-d-maltoside are colored in green, orange, and purple, respectively. (E) Overlay of the backbone traces for the six FtsX_ECL1_ crystal structures (colored in green, orange, and purple in the presence of dodecyltrimethylammonium chloride, *n*-undecyl-β-d-maltoside, and *n*-decyl-β-d-maltoside, respectively) with the backbone traces of the 20 FtsX_ECL1_ NMR conformers (in gray). (F) Cartoon representation of the FtsX_ECL1_ crystallographic structure (in cyan) overlaid on the M. tuberculosis FtsX_ECD_ structure (in orange) (PDB code 4N8N). Cysteine residues involved in disulfide bond formation are shown as yellow sticks and indicated with an arrow. (G) Structure-based sequence alignment (54) of extracellular domains from S. pneumoniae (*Spn* ECL1) (PDB code 6HFX, chain A) and M. tuberculosis (*Mtb* ECD) (PDB code 4N8N, chain B). Secondary structure elements from each protein are indicated and numbered.

The three-dimensional structure of FtsX_ECL1_ (residues 46 to 168) was solved by both NMR spectroscopy (Fig. 1A) and X-ray crystallography (Fig. 1B). The folded structure (residues 57 to 166) reveals a central core composed of a four-stranded antiparallel β-sheet (β1, β6, β4, and β5) and two helices (α1 and α3), an α-helical lobe (residues 107 to 135) harboring the α2 helix, and an extended β-hairpin (β2 and β3). The β-hairpin and helical lobes are connected to the central core by hinges. Details for structural determination of FtsX_ECL1_ by NMR in the absence of detergent are presented in Materials and Methods, and structure statistics are summarized in Table 1. The solution structure shows that while the central mixed α-β core adopts a well-defined conformation, the two appended lobes are highly dynamic on multiple timescales (see below), presenting a range of conformations among the 20 members of the FtsX_ECL1_ NMR structural ensemble (Fig. 1A).

**TABLE 1:** Structural statistics for NMR solution structure of FtsX_ECL1_^*a*^

Parameter	Value
NMR distance and angle restraints	
Total NOE-based distance constraints	1,711
Intraresidue (*i* = *j*)	232
Sequential (|*i* − *j*| = 1)	499
Medium range (1 < |*i* − *j*| < 5)	365
Long range (|*i* − *j*| ≥ 4)	615
Maximum distance violation (Å)	0.49
Dihedral angle restraints	110
Maximum dihedral angle violation (°)	7
Total number of RDCs used (measured)	82 (112)
Q factor	0.11 (0.39)
Correlation (experimental to calculated)	0.99 (0.89)

RMS deviations from idealized covalent geometry	
Covalent bond lengths (Å)	0.011
Covalent angle values (°)	1.3

Ramachandran analysis (%)^*b*^^,^^*c*^	
Most favored	86.5
Additionally allowed	8.4
Generously allowed	1.8
Disallowed	3.3

RMSD values (Å)	
Backbone atoms (all)	1.5
All heavy atoms (all)	1.9
Backbone atoms (core^*b*^)	0.4
All heavy atoms (core^*b*^)	0.9

a From the ensemble of the 20 best NMR structures. PDB code 6MK7; BMRB code 30523.

b Calculated over the core of the structure, excluding the helical lobe and β-hairpin, residues 59 to 65, 89 to 108, and 134 to 165.

c As computed by Procheck.

Three different structures, with resolutions ranging from 2.0 to 2.3 Å, were solved by X-ray crystallography, each in the presence of a different detergent. The detergents used were dodecyltrimethylammonium chloride (detergent 1 [D1]), *n*-undecyl-β-d-maltoside (detergent 2 [D2]), and *n*-decyl-β-d-maltoside (detergent 3 [D3]). The presence of detergents was critical, as in their absence, crystals diffracted at very low resolution (≤4 Å), suggesting significant mobility in some protein regions. Details from the crystallographic determination are provided in Materials and Methods, and structure statistics are summarized in Table 2. In all cases, two independent protein molecules are present in the asymmetric unit (Fig. S1 in the supplemental material), arranged in such a way as to keep hydrophobic residues from their α-helical lobes buried. As explained below, the α-helical-lobe region is relevant for PcsB interaction. In total, six independent structures were determined for both the protein structure and associated detergent molecules (Fig. S2). Different conformations were observed for the β-hairpin and the helical lobe among the six structures, depending on the presence and identity of the detergent molecule bound to FtsX_ECL1_ (Fig. S1B). The structural variations observed in these crystallographic structures, however, are less dramatic than those observed in the NMR conformer bundle obtained in the absence of ligand (Fig. 1E). As expected, regions presenting structural variations in crystal structures correspond to those showing a highly dynamic behavior by NMR.

**TABLE 2:** Crystallographic data collection and refinement statistics

Parameter	Values for^*a*^:		
	FtsX_ECL1_-D1	FtsX_ECL1_-D2	FtsX_ECL1_-D3
Data collection statistics			
Wavelength (Å)	0.97934	0.97934	0.97934
Space group	P 4_3_ 2_1_ 2	P 4_3_ 2_1_ 2	P 4_3_ 2_1_ 2
Unit cell dimensions			
*a*, *b*, *c* (Å)	75.87, 75.87, 97.93	84.61, 84.61, 106.19	75.49, 75.49, 95.31
α, β, γ (º)	90, 90, 90	90, 90, 90	90, 90, 90
Temp (K)	100	100	100
X-ray source	Synchrotron	Synchrotron	Synchrotron
Resolution range (Å)	47.05–(2.05–2.0)	44.97–(2.38–2.3)	46.58–(2.23–2.16)
No. of unique reflections	19,992 (1,436)	17,781 (1,700)	15,360 (1,496)
Completeness (%)	100 (100)	100 (100)	99.85 (99.93)
Multiplicity	24.4 (25.9)	22.1 (22.5)	16.6 (21.8)
*R*_pim_^*b*^	0.013 (0.535)	0.007 (0.123)	0.010 (0.241)
*<I/σ(I)>*	21.9 (1.5)	41.7 (4.6)	24.6 (2.55)
CC1/2^*c*^	1.00 (0.67)	1.00 (0.98)	1.00 (0.89)

Refinement statistics			
Resolution range (Å)	41.14–2.0	42.31–2.3	46.58–2.16
*R*_work_/*R*_free_^*d*^	0.24/0.28	0.26/0.30	0.25/0.31
No. of atoms			
Protein	1,888	1,732	1,856
Water	25	21	23
Ligand	16	47	33

RMS deviations			
Bond length (Å)	0.008	0.009	0.010
Bond angles (°)	1.27	1.22	1.29

Ramachandran plot			
Favored/outlier regions (%)	95.65/0.43	93.20/2.43	92.41/0.00
Monomers per AU	2	2	2

PDB code	6HE6	6HEE	6HFX

a Values between parentheses correspond to the highest-resolution shells.

b *R*_pim_ measures the precision of averaged intensities. *R*_pim_ = Σ*_hkl_*[1/(*N* − 1)] 1/2 Σ*_i_* | *I_i_*(*hkl*) − [*I*(*hkl*)] | /Σ*_hkl_*Σ*_i_I_i_*(*hkl*), where Σ*_i_I_i_*(*hkl*) is the *i*th measurement of reflection *hkl*, [*I*(*hkl*)] is the weighted mean of all measurements, and *N* is the redundancy for the *hkl* reflection.

c CC1/2 is the correlation coefficient between intensity estimates from half data sets.

d *R*_work_/*R*_free_ = Σ*_hkl_*| *F_o_* − *F_c_* | /Σ*_hkl_* | *F_o_* |, where *F_c_* is the calculated and *F_o_* is the observed structure factor amplitude of reflection *hkl* for the working/free (5%) set, respectively.

10.1128/mBio.02622-18.1 FIG S1 FtsX_ECL1_ crystal structures solved in this work. (A) Cartoon representation of the asymmetric unit of the FtsX_ECL1_-D1 crystal structure, with chain B colored blue to red from the N terminus to the C terminus of the domain, and chain A shown in gray. The detergent 1 molecule (D1) is represented as sticks. (B) Structural superposition of the six independent crystal structures of FtsX_ECL1_ solved in this work. All chains are presented with a ligand attached, except for chain B in complexes FtsX_ECL1_-D1 and FtsX_ECL1_-D3. (C) Structural superposition of the six independent structures. Color coding is as described for panel B. (D) Polar interactions in the N-terminal region of FtsX_ECL1_-D1 (chain A). The N-terminal region (residues 49 to 59) is represented as sticks. Polar interactions are represented as dashed lines. (E) Stabilization of detergent 2 (D2) by the helical lobe of FtsX_ECL1_. FtsX_ECL1_-D2 complex. *n*-Undecyl-β-d-maltoside (D2) is represented as white sticks. Relevant residues in the protein are represented as green sticks. (F) Stabilization of detergent 3 (D3) by the helical lobe of FtsX_ECL1_. FtsX_ECL1_-D3 complex. *n*-Decyl-β-d-maltoside (D3) is represented as white sticks. Relevant residues in the protein are represented as green sticks. Polar interactions are represented by dashed lines. (G) Full stabilization of the detergent tail in FtsX_ECL1_-D3 complex (green ribbon) requires hydrophobic residues from the helical lobe of a second monomer in the crystal (yellow ribbon). Similar packing was observed for the other complexes. Download FIG S1, EPS file, 13.2 MB. Copyright © 2019 Rued et al. 2019 Rued et al. This content is distributed under the terms of the Creative Commons Attribution 4.0 International license.

10.1128/mBio.02622-18.2 FIG S2 Electron density maps for FtsX_ECL1_. (A) Electron density map for the 2.0-Å-resolution structure of FtsX_ECL1_ monomer (chain A). (B) Electron density map (2*F_o_* − *F_c_* map contoured at 0.8σ) for detergent D1 (dodecyltrimethylammonium chloride). (C) Electron density map (2*F_o_* − *F_c_* map contoured at 1σ) for detergent D2 (*n*-undecyl-β-d-maltoside). (D) Electron density map (2*F_o_* − *F_c_* map contoured at 1σ) for detergent D3 (*n*-decyl-β-d-maltoside). Detergents are represented as sticks. Download FIG S2, EPS file, 2.9 MB. Copyright © 2019 Rued et al. 2019 Rued et al. This content is distributed under the terms of the Creative Commons Attribution 4.0 International license.

In any case, the crystal structures suggest that changes in the protein backbone and side chains of the helical lobe occur when a detergent ligand is bound (Fig. 1C). These changes create a cavity in which the detergent molecules insert (Fig. 1D), with a large part of the helical lobe (from Q111 to E127) affected by the interaction with detergent (Fig. 1C). Residues Y112, W123, E127, and F126 are strongly perturbed upon detergent ligand binding (Fig. 1C), with the hydroxyl moiety of Y112 interacting with carboxylate of E127. Changes in W123 and F126 stabilize the hydrophobic region of detergent 1. A similar interaction pattern is observed for detergents 2 and 3; additional interactions are observed in N131 with detergents 2 and 3, which are characterized by a larger hydrophilic head (maltose) (Fig. S1E and F). Full stabilization of the hydrophobic tail of the detergents is completed by the same hydrophobic residues (W123 and F126) but from a symmetry-related molecule (Fig. S1G). Although a physiological role for detergent binding to the helical lobe is unknown, many of these same residues are important for the interaction with PcsB (see below).

A structural comparison with the M. tuberculosis FtsX_ECL1_ (PDB code 4N8N) (16) reveals differences in both the overall structure (root mean square deviation [RMSD] of 2.2 Å) and the appended lobes of the core domain (Fig. 1F). The main differences between the mycobacterial and pneumococcal FtsX_ECL1_ domains are the presence of an extra helix (α1) and a disulfide bond in the M. tuberculosis ECL1 that are absent in the pneumococcal ECL1 and an extended β-hairpin (residues 71 to 87) that is unique to the pneumococcal ECL1 domain. Of note, this β-hairpin was conserved among aligned streptococcal species sequences (see Fig. S7A for species list), with β3 being more conserved than β2. It is also worth noting that in the M. tuberculosis ECL1, the upper and lower lobes form a large hydrophobic cleft with four exposed Phe residues (F61, F110, F113, and F122), and this region was suggested as a strong candidate for the interaction surface between FtsX and PG hydrolase RipC (16). These phenylalanine residues are not conserved in the pneumococcal ECL1 (Fig. 1F and G), but the hydrophobic nature of this region is preserved (Y63, I118, W123, and L133).

### FtsX_ECL1_ is dynamic in solution.

We also performed additional NMR experiments to explore the mobility of the FtsX_ECL1_ domain, both to validate the heterogeneity of the structural ensemble in solution and to elucidate function. The ^1^*D*_HN_ residual dipolar couplings (RDCs) obtained by weak alignment in *Pf*1 filamentous phage correspond well to previously determined secondary structure elements (29), with uniform values for the entire length of α1 and α2, as expected for straight helices. In contrast, ^1^*D*_HN_ values near 0 for the N-terminal tail, the very C terminus, and the nonhelical part of the helical lobe between α2 and β5 are suggestive of significant conformational disorder in solution (Fig. S3A). These regions of small or zero RDCs are regions of very high RMSD in the NMR structure bundle (Fig. S3B). As anticipated, the correlation between experimentally measured RDCs and predicted RDCs back-calculated from the structures (30) is high, but only in the core subdomain, and the experimental and predicted RDCs match poorly in the β-hairpin for most of the crystal structures (Fig. S3C and D).

10.1128/mBio.02622-18.3 FIG S3 Dynamics of FtsX_ECL1_ in solution and crystal. (A) ^1^D_HN_ residual dipolar couplings (RDCs) measured in *Pf*1 filamentous phage, plotted against the FtsX_ECL1_ sequence. (B) Cα RMSD of the solution structure, mapped onto a representative structure from the NMR ensemble, with increased RMSD shown as increased tube width and redness. (C) Box-and-whisker plot showing the distribution of differences between measured and predicted values for the NMR structure for all measured RDCs, including those that were not used as restraints. Outliers are shown as circles. The dashed line shows the median difference for all NMR structures, and the gray-shaded region contains 95% of difference values for all NMR structures, excluding lightly shaded hypermobile regions with vanishing RDC values (residues 45 to 58, 119 to 133, and 165 to 168). Red X’s mark difference values for the crystal structure with detergent D1. Secondary structure elements are shown above for reference. (D) Average RDC difference values for the crystal structures with D1 mapped onto the structure, with excluded hypermobile regions shown in black. (E) Heteronuclear NOEs (hNOE) for each residue. Secondary structure elements are shown in inset for reference. (F) hNOE values mapped onto a representative structure. Increasing fast-timescale motions are shown with increasing redness and increasing tube thickness. Residues omitted due to peak overlap or absence are shown in black in panels D, F, H, and J. (G) Nuclear spin relaxation rates: *R*_2_/*R*_1_ for each residue. (H) *R*_2_/*R*_1_ mapped onto a representative structure, with elevated values indicative of slow-timescale motions shown in blue and decreased values indicative of fast-timescale motions shown in red. (I) *R*_ex_ exchange rates for each residue, at 600 MHz and 800 MHz plotted in black and gray, respectively. (J) *R*_ex_ rates at 600 MHz mapped onto a representative structure, with increasing slow-timescale motions shown with increasing blueness and increasing tube thickness. (K) Crystallographic B factors plotted as increasing tube width and redness on the FtsX_ECL1_ structure, for chains A and B with detergents D1 and D3. The detergents are shown as sticks. (L) RMSD of the crystal structures mapped onto chain A of the FtsX_ECL1_-D1 structure, with increasing tube thickness and redness indicative of higher RMSDs. Download FIG S3, EPS file, 8.8 MB. Copyright © 2019 Rued et al. 2019 Rued et al. This content is distributed under the terms of the Creative Commons Attribution 4.0 International license.

We previously reported that the ^15^N-{^1^H} heteronuclear nuclear Overhauser effect (hNOE) is low or negative at the termini, indicating that they are highly flexible in solution (29). The hNOE is also smaller in the β2-β3 hairpin region, as well as in the C-terminal end of the α2 helix and the subsequent coiled region leading into β5 (Fig. S3E) (29). Mapping these dynamics data onto a representative structure from the solution NMR ensemble shows that these regions with fast-timescale dynamics correspond to the β-hairpin and the helical lobe (Fig. S3F). Information on picosecond-to nanosecond (ps-ns) fast-timescale motions extracted from the *R*_2_/*R*_1_ ratio also reveals that the α2-β5 linker is highly flexible, while the β1-β2 linker and β-hairpin show elevated *R*_2_/*R*_1_ ratios, specifically indicative of slow, millisecond (ms) timescale conformational exchange (Fig. S3G and H). These findings were directly confirmed by Carr-Purcell-Meiboom-Gill (CPMG) relaxation dispersion NMR spectroscopy (Fig. S3I and J) (31). We conclude that the β-hairpin exhibits flexibility on both the sub-ns and ms timescales. Interestingly, the β1-β2 linker also shows increased B factors that are qualitatively consistent with the ms timescale conformational exchange observed by NMR.

These complex motions observed in the solution dynamics experiments are reflected in the heterogeneity of the NMR structure, with high Cα RMSDs particularly in the α2-β5 linker but also in the β-hairpin and β1-β2 linker, thus validating the conformational spread in the ECL1 structure in solution (Fig. S3B). The dynamic nature of the helical lobe is also reflected in the heterogeneity and B factors of the crystal structures (Fig. S3K and L). Full-length FtsX itself is likely a dimer *in vivo* (32), and one can speculate that the flexible helical-lobe and β-hairpin regions may contribute to dimerization or to interactions with a binding partner like PcsB.

### The PscB_CC_ domain interacts with FtsX_ECL1_.

The ^1^H-^15^N HSQC spectrum for ECL1 has excellent chemical shift dispersion and lends itself readily to studies of protein-protein interactions (Fig. S4A). In contrast, full-length PcsB is 42 kDa and forms a dimer and thus is challenging to study by NMR due to its size. We therefore constructed truncation mutants of PcsB, focusing on the coiled-coil domain (PscB_CC_), thereby limiting the molecular weight to 23 to 24 kDa. ^15^N-labeled PscB_CC_(47–267) (comprising residues 47 to 267) and PscB_CC_(47–254) (comprising residues 47 to 254) are both characterized by ^1^H-^15^N HSQC spectra with limited ^1^H signal dispersion (Fig. S6A), consistent with the high helical content of this domain. Circular dichroism spectroscopy confirms that these proteins are primarily α-helical, in agreement with the crystal structure (22), indicating that they are properly folded (Fig. S6A) and can be used for ECL1 binding studies.

10.1128/mBio.02622-18.4 FIG S4 NMR chemical shift perturbation experiments demonstrate that the PcsB coiled-coil domain interacts with FtsX_ECL1_. (A) ^1^H-^15^N HSQC titration of 50 µM ^15^N-labeled FtsX_ECL1_ with molar equivalents of unlabeled PcsB coiled-coil domain [PscBCC(47–267)]. The legend beside the ^1^H-^15^N HSQC spectra indicates the amount of PscB_CC_ added and the spectrum peak color corresponding to this amount. Red peaks indicate no PscB_CC_ added, dark blue peaks indicate 1 molar equivalent PscB_CC_ added, light blue peaks indicate 2 molar equivalents PscB_CC_ added, dark purple peaks indicate 4 molar equivalents PscB_CC_ added, light purple peaks indicate 6 molar equivalents PscB_CC_ added. Labeled peaks on the ^1^H-^15^N HSQC indicate residues shifting more than one or two standard deviations from the mean value when 2 molar equivalents of PscB_CC_ were added. (B) Zoomed depiction of residues F126 and M119 from ^1^H-^15^N HSQC titration of ^15^N-labeled FtsX_ECL1_ with unlabeled PscB_CC_, as examples of peaks with significant shifts (see panel C). The legend to panel A indicates the amount of PscB_CC_ added and the spectrum peak color corresponding to this amount. (C) Chemical shift (Δδ, ppm) per residue of FtsX_ECL1_ upon the addition of 2 molar equivalents of PscB_CC_ is shown in red. The secondary structure is indicated above the plot. The black line on the plot (2 SD) represents the threshold of 2 standard deviations from the average chemical shift upon the addition of 2 equivalents of PscB_CC_. Peak height change before and after addition of 2 molar equivalents of PscB_CC_ to FtsX_ECL1_ is shown in blue. Download FIG S4, EPS file, 4.3 MB. Copyright © 2019 Rued et al. 2019 Rued et al. This content is distributed under the terms of the Creative Commons Attribution 4.0 International license.

**FIG 2:**
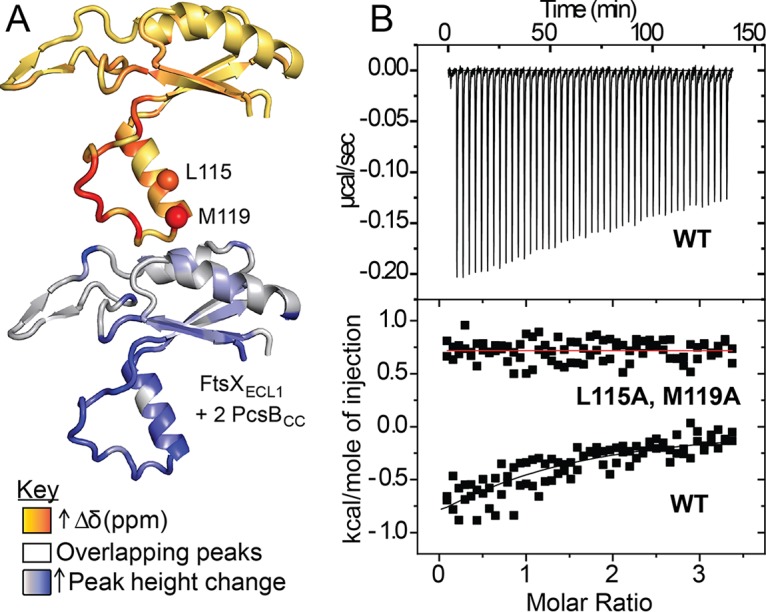
FtsX_ECL1_ binds PscB_CC_. (A) Significant chemical shifts and peak height changes upon ^1^H-^15^N HSQC titration of 50 µM ^15^N FtsX_ECL1_ with 100 µM unlabeled PscB_CC_ map to the lower lobe of FtsX_ECL1_. Chemical shifts (Δδ, ppm) and peak height changes are mapped as color gradients on the FtsX_ECL1_ structure, orange to red and light gray to blue, respectively. L115 and M119 α carbons are shown as spheres on the upper image. Peaks that overlap in the ^1^H-^15^N HSQC spectra are colored white. Chemical shifts and peak height changes upon the addition of 2 molar equivalents of PscB_CC_ to FtsX_ECL1_ are mapped to the structure. (B) Representative titration of PscB_CC_ with wild-type (WT) FtsX_ECL1_ or L115A, M119A FtsX_ECL1_ as monitored by ITC. Conditions used were 50 mM potassium phosphate, 50 mM NaCl, 0.5 mM EDTA, pH 7.0, at 25.0°C. Top, corrected ITC data; bottom, kcal/mole of injection versus time. The black line overlapping the WT data indicates the best fit to a one-site binding model. Fitting parameters are summarized in Table 3. The red line drawn through the L115A M119A FtsX_ECL1_ data is for reference.

To determine if PcsB physically interacts directly with FtsX_ECL1_, we titrated unlabeled PscB_CC_(47–267) into ^15^N-labeled FtsX_ECL1_ at molar ratios of 1:1, 2:1, 4:1, and 6:1 and recorded the ^1^H-^15^N HSQC spectra (Fig. S4A and B). Numerous crosspeaks move in response to the addition of PscB_CC_ to ^15^N-labeled FtsX_ECL1_. The largest changes occur in the helical lobe of FtsX_ECL1_ (Fig. 2A, Fig. S4 and S5). In particular, residues M119, W123, I125, F126, and G128 exhibit the greatest changes in crosspeak position and intensity in the sample with a 1:2 molar ratio of FtsX_ECL1_ to PscB_CC_ (Fig. 2A, Fig. S4C); when additional PscB_CC_ is added, these crosspeaks broaden beyond detection (Fig. S5B and C). A reciprocal ^1^H-^15^N HSQC spectroscopy experiment with ^15^N-labeled PscB_CC_(47–254) further confirms an interaction with unlabeled FtsX_ECL1_ (Fig. S6B), as multiple crosspeaks shift upon the addition of increasing amounts of FtsX_ECL1_ (Fig. S6B). We measured the binding affinity of the FtsX_ECL1_–PscB_CC_ complex using isothermal titration calorimetry (ITC), which reveals an association equilibrium constant (*K_a_*) of 3.0 × 10^4^ M^−1^ (*K_d_* ∼ 34 µM) (Table 3, Fig. 2B). These data provide the first biochemical evidence for a direct physical interaction between PscB_CC_ and FtsX_ECL1_ (Fig. 2, Fig. S4 to S6).

**TABLE 3:** Thermodynamic parameters of wild-type and mutant FtsX_ECL1_ from direct analysis of isothermal titration calorimetry^*a*^

Protein	*K_a_* (×10^4^ M^−1^)	*K_d_* (µM)^*b*^	Δ*H* (kcal mol^−1^)	Δ*S* (kcal mol^−1^ K^−1^)
FtsX_ECL1_	3.0 ± 0.5	34 ± 6	−2.1 ± 0.2	13.4
L115A M119A FtsX_ECL1_	NA^*c*^	NA^*c*^	NA^*c*^	NA^*c*^

a Solution conditions were 50 mM sodium phosphate, 50 mM NaCl, 0.5 mM EDTA, pH 7.0, at 25°C. The chemical model used to fit the data is indicated in supplemental materials and methods in Text S1.

b *K_D_*, equilibrium dissociation constant.

c NA, not applicable since no heat was observed.

10.1128/mBio.02622-18.5 FIG S5 Significant chemical shift changes and peak height changes upon ^1^H-^15^N HSQC titration of 50 µM ^15^N-labeled FtsX_ECL1_ with unlabeled PscB_CC_ map to the lower lobe of FtsX_ECL1_. Chemical shift changes (Δδ, ppm) and peak height changes are mapped as color gradients on the FtsX_ECL1_ structure, orange to red and grey to blue, respectively. Peaks that overlap or disappear in the ^1^H-^15^N HSQC spectra are colored white and dark grey, respectively. (A) Chemical shift changes and peak height changes upon the addition of 1 molar equivalent of PscB_CC_ to FtsX_ECL1_. (B) Chemical shift changes and peak height changes upon the addition of 4 molar equivalents of PscB_CC_ to FtsX_ECL1_. (C) Chemical shift changes and peak height changes upon the addition of 6 molar equivalents of PscB_CC_ to FtsX_ECL1_. Download FIG S5, EPS file, 8.5 MB. Copyright © 2019 Rued et al. 2019 Rued et al. This content is distributed under the terms of the Creative Commons Attribution 4.0 International license.

10.1128/mBio.02622-18.6 FIG S6 PscB_CC_ binds to FtsX_ECL1_. (A) Left, ^1^H-^15^N HSQC spectrum of ^15^N-labeled PscB_CC_(47–254) (in red) overlaid with ^1^H-^15^N HSQC spectrum of ^15^N-labeled PcsB-CC(47–267) (in blue). Right, circular dichroism (CD) spectra of His-PscB_CC_(47–267) (in blue) and His-PscB_CC_(47–254) (in red). Primarily α-helical secondary structure is observed. (B) Left, ^1^H-^15^N HSQC titration of 50 µM ^15^N-labeled PscB_CC_(47–254) with molar equivalents of unlabeled FtsX_ECL1_. The legend above the ^1^H-^15^N HSQC spectra indicates the amount of FtsX_ECL1_ added and the spectra peak color corresponding to this amount. Red peaks indicate no FtsX_ECL1_ was added, dark blue peaks indicate 1 molar equivalent FtsX_ECL1_ was added, light blue peaks indicate 2 molar equivalents FtsX_ECL1_ was added, and gold peaks indicate 4 molar equivalents FtsX_ECL1_ was added. Right, portions of the ^1^H-^15^N HSQC spectra of ^15^N-labeled PscB_CC_(47–254) that have peak shifts upon the addition of increasing amounts of FtsX_ECL1_. Download FIG S6, EPS file, 8.1 MB. Copyright © 2019 Rued et al. 2019 Rued et al. This content is distributed under the terms of the Creative Commons Attribution 4.0 International license.

10.1128/mBio.02622-18.11 TEXT S1 Supplemental materials and methods and appendix. Download Text S1, PDF file, 2.6 MB. Copyright © 2019 Rued et al. 2019 Rued et al. This content is distributed under the terms of the Creative Commons Attribution 4.0 International license.

### The interaction region of FtsX_ECL1_ with PscB_CC_ is essential for cell growth and proper morphology.

Having identified the interaction region between FtsX_ECL1_ and PscB_CC_, we next sought to determine the degree to which this interaction interface mapped by NMR spectroscopy contributes to pneumococcal viability. A multiple-sequence alignment of this region (residues 102 to 155) among bacterial species in which FtsX has been studied and in related streptococcal species (Fig. S7A) reveals that amino acids in this region are either partially or completely conserved (Fig. S7A). We therefore decided to target E109, Q111, L115, M119, W123, F126, and N131 for substitution with alanine, singly or in combination (Fig. S7A). Given the essentiality of the interaction of FtsX_ECL1_ and PscB_CC_, we predicted that mutating these residues might be lethal (20). To allow for the cross-in of potentially lethal point mutations, we employed the Janus cassette method to insert point mutations at the native site of *ftsX* into a strain containing an ectopic copy of *ftsX^+^* under a zinc-inducible promoter (Fig. S7B) (33). We then transformed markerless mutant alleles of *ftsX* in the presence of zinc. This allows for expression of the wild-type *ftsX^+^* and mutant *ftsX* simultaneously. As long as the mutant *ftsX* was not dominant negative, we could obtain a strain that expresses the wild-type copy of *ftsX^+^* under zinc induction and mutant *ftsX* only in the absence of zinc (Fig. S7B).

10.1128/mBio.02622-18.7 FIG S7 Residues in the interaction region of FtsX_ECL1_-PcsB_CC_ are conserved, and FtsX depletion results in rounded cells and inhibition of growth. (A) Amino acid alignment of residues 102 to 155 of *S. pneumoniae* FtsX_ECL1_ with corresponding FtsX sequences from *M. tuberculosis*, *E. coli*, *Streptococcus gordonii*, *Streptococcus mitis*, *Streptococcus mutans*, *Streptococcus pyogenes*, *Bacillus cereus*, and *B. subtilis*. The *S. pneumoniae* sequence is indicated in bold, and the secondary structure from the *S. pneumoniae* structure is indicated above the alignment. The FtsX_ECL1_-PcsB_CC_ interaction region is indicated by the blue line beneath the alignment, from *S. pneumoniae* FtsX amino acids 105 to 137. Alignment was obtained by input of the sequences into the Clustal Omega webserver (http://www.ebi.ac.uk/Tools/msa/clustalo/) and then input of the multiple sequence alignment into the ESPript 3.0 webserver (http://espript.ibcp.fr/ESPript/ESPript/). See supplemental materials and methods in Text S1 for additional details. (B) Genetic scheme for cross-in of potentially lethal amino acid changes. In the D39 *rpsL1* Δ*cps* wild-type background, wild-type *ftsX* was placed under the control of the zinc-inducible promoter (P_Zn_) at the *bgaA* locus (ectopic site) and a Janus cassette (33) [*kan-rpsL*^+^]-*ftsX* was placed at the native site of *ftsX* in the chromosome (D39 *rpsL1* Δ*cps* [*kan-rpsL*^+^]-*ftsX*/*bgaA*::*tet*-P_Zn_-*ftsX^+^*; strain IU12330). To select for the cross-in of markerless mutant alleles of *ftsX* that could potentially be lethal, linear DNA amplicons of mutant *ftsX* were transformed into IU12330 with 0.45 mM ZnCl_2_, 0.045 mM MnSO_4_, and streptomycin (for Janus cassette exchange). Resulting strains were propagated and stored with the same Zn and Mn concentrations. After storage, strains were grown without Zn-Mn to determine if the mutant allele of *ftsX* caused morphological or growth defects. Strains were also grown with Zn-Mn to determine if the mutant allele was dominant negative. (C) Representative growth curves of the FtsX depletion strain versus the wild type. These strains were grown with or without 0.45 mM ZnCl_2_ supplemented with 0.045 mM MnSO_4_ (indicated as +Zn). Strains shown are as follows: black circle, D39 *rpsL1* Δ*cps* wild-type parent (1, strain IU1824); grey circle, IU1824 +Zn; blue square, D39 *rpsL1* Δ*cps* Δ*ftsX*::P-*aad9*/*bgaA*::*tet-*P_Zn_*-ftsX^+^* (2, strain IU12376); light blue square, IU12376 +Zn. This growth curve experiment was repeated three times with similar results. (D) Representative images of strains at 6 h of growth. The genotype of the strain shown is indicated above each panel. No Zn or +Zn indicates whether or not Zn-Mn was added. %, the percentage of cells in the population that are morphologically similar to the images shown. More than 50 cells were counted per experimental run, condition, and strain. These experiments were performed three times independently with similar results. Scale bar shown is 1 µm. (E) Lengths, widths, aspect ratios, and relative cell volumes of strains at 3 h and 6 h of growth. Strains are indicated according to the numbering in panel C. More than 50 cells were measured per strain and condition over two experimental replicates. For statistical analysis, the Kruskal-Wallis test (one-way ANOVA) with Dunn’s multiple comparison posttest was used to determine if lengths, widths, aspect ratios, and relative cell volumes were significantly different between strains and conditions. ns, not significant; *, *P* < 0.05; ** *P* < 0.005; ***, *P* < 0.0005. Download FIG S7, EPS file, 6.2 MB. Copyright © 2019 Rued et al. 2019 Rued et al. This content is distributed under the terms of the Creative Commons Attribution 4.0 International license.

Zinc toxicity has been observed to cause aberrant cell morphology and growth inhibition in S. pneumoniae when cells are not supplemented with manganese (34, 35). To rule out any deleterious effects of the zinc-and-manganese (Zn-Mn) addition used to induce *ftsX* expression, we measured the growth of the parent and the FtsX merodiploid strain in the presence of these metals. To verify that the addition of 0.45 mM ZnCl_2_ and 0.045 mM MnSO_4_ (Zn-Mn) did not cause growth or morphological defects, cells were grown in the presence and absence of Zn-Mn and imaged at 3 h and 6 h into the growth curve (Fig. S7C and D). Wild-type cells (strain D39 Δ*cps rpsL1*) had no morphological or growth defects at these time points with or without the addition of Zn-Mn (Fig. S7C and E).

In contrast, the FtsX merodiploid strain (P_Zn_-*ftsX* Δ*ftsX*) had significant morphological and growth defects at 3 or 6 h in the absence of Zn-Mn (Fig. S7C to E). Cessation of growth and aberrant cell morphology were observed in 90% of cells at 3 h and 95% of cells at 6 h growth (Fig. S7D). These cells were significantly shorter and rounder than wild-type cells (Fig. S7E), and a large variability in their volumes was observed (Fig. S7E), as previously found for a strain expressing *ftsX^+^* under a fucose-inducible promoter (21). If the strain was grown in the presence of Zn-Mn, FtsX was expressed and the strain had no growth or morphological defects (Fig. S7C to E). This indicates the defects observed were solely due to the absence of FtsX.

**FIG 3:**
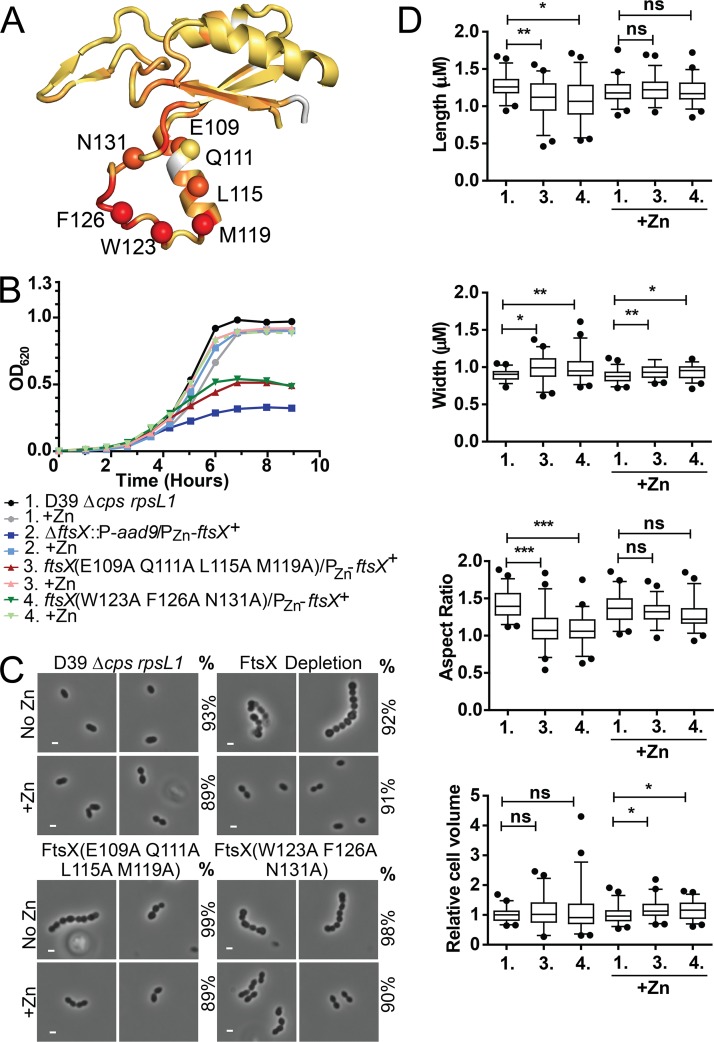
Multiple amino acid changes in the lower lobe of FtsX_ECL1_ cause morphological and growth defects. (A) Amino acid changes made are mapped to the structure of FtsX_ECL1_. The α carbon of each residue is shown as a colored sphere on the structure, and the orange-to-red coloring represents the peak height changes in ^1^H-^15^N HSQC spectra upon the addition of 2 molar equivalents of PscB_CC_ to FtsX_ECL1_. (B) Representative growth curves of strains with amino acid changes in the lower lobe of FtsX_ECL1_ compared to the growth of an FtsX depletion strain. These strains were grown without or with 0.45 mM ZnCl_2_ with 0.045 mM MnSO_4_ (Zn-Mn; indicated as +Zn). Strains shown are as follows: black circle, strain D39 *rpsL1* Δ*cps* wild-type parent (1, strain IU1824); gray circle, IU1824 +Zn; blue square, D39 *rpsL1* Δ*cps* Δ*ftsX*::P-*aad9*/*bgaA*::*tet-*P_Zn_*-ftsX^+^* (2, strain IU12376); light blue square, IU12376 +Zn; red triangle, D39 *rpsL1* Δ*cps ftsX*(E109A Q111A L115A M119A)/*bgaA*::*tet-*P_Zn_*-ftsX^+^* (3, strain IU12861); pink triangle, IU12861 +Zn; green inverted triangle, D39 *rpsL1* Δ*cps ftsX*(F126A W123A N131A)/*bgaA*::*tet-*P_Zn_*-ftsX^+^* (4, strain IU12864); light green inverted triangle, IU12864 +Zn. This growth curve experiment was repeated three times with similar results. (C) Representative images of strains at 6 h of growth. The genotype or phenotype of the strain is indicated above each panel. No Zn, Zn-Mn was not added; +Zn, Zn-Mn was added; %, percentage of cells in the population that are morphologically similar to the images shown. More than 50 cells per strain, condition, and experimental repeat were analyzed. These experiments were performed three times independently with similar results. Scale bar shown is equal to 1 µm. (D) Lengths, widths, aspect ratios, and relative cell volumes of strains at 6 h of growth. Strains are indicated according to numbering in panel B. More than 50 cells were measured per strain and condition over two experimental replicates. For statistical analysis, the Kruskal-Wallis test (one-way ANOVA) with Dunn’s multiple-comparison posttest was used to determine if lengths, widths, aspect ratios, and relative cell volumes were significantly different between strains and conditions. ns, not significant; *, *P* < 0.05; **, *P* < 0.005; ***, *P* < 0.0005.

**FIG 4:**
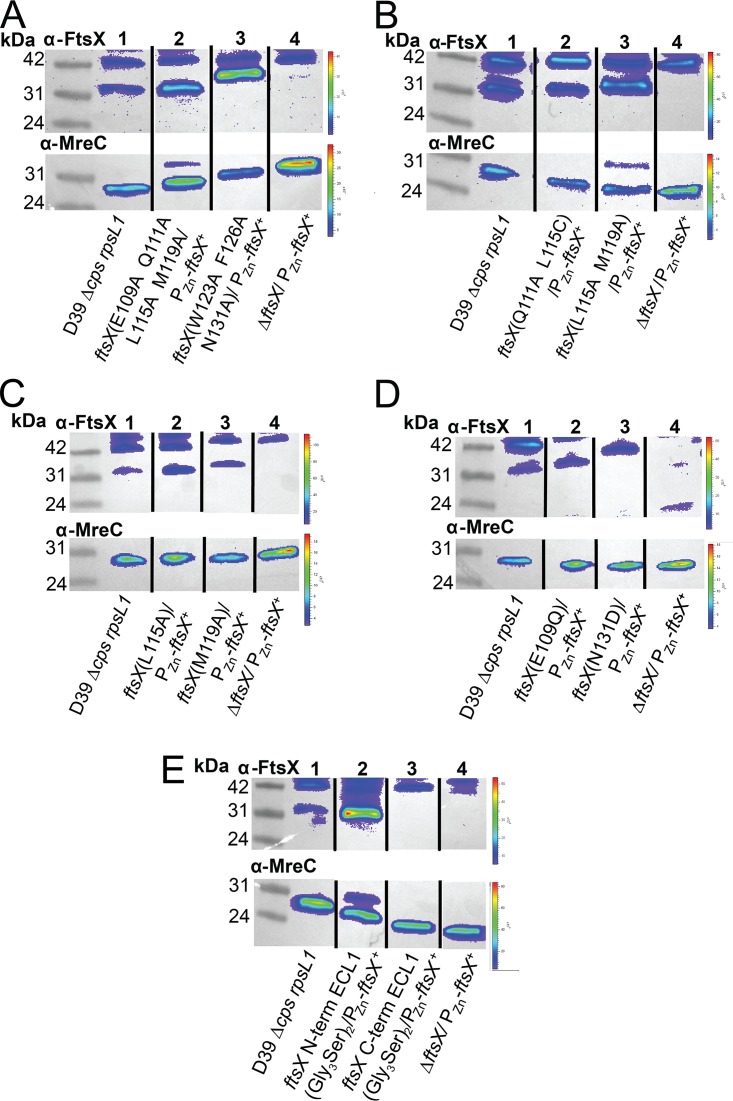
FtsX mutants with amino acid changes are expressed at nearly wild-type levels. Representative blots of anti-FtsX and anti-MreC (Western blotting control for loading) antibodies are shown, with the genotype indicated under each lane. Expected molecular weight (MW) for FtsX is 34.2 kDa, and expected MW for MreC is 29.7 kDa. Samples were grown without Zn and harvested at 6 h of growth. Western blots were imaged as described in supplemental materials and methods in Text S1. (A) FtsX(E109A Q111A L115A M119A) and FtsX(W123A F126A N131A) are expressed at or above wild-type levels without zinc. Lane 1, D39 *rpsL1* Δ*cps* (strain IU1824); lane 2, D39 *rpsL1* Δ*cps ftsX*(E109A Q111A L115A M119A)/*bgaA*::*tet-*P_Zn_*-ftsX^+^* (strain IU12861); lane 3, D39 *rpsL1* Δ*cps ftsX*(W123A F126A N131A)/*bgaA*::*tet-*P_Zn_*-ftsX^+^* (strain IU12864); lane 4, D39 *rpsL1* Δ*cps* Δ*ftsX*/*bgaA*::*tet-*P_Zn_*-ftsX^+^* (strain IU13461). (B) FtsX(Q111A L115C) and FtsX(L115A M119A) are expressed at nearly wild-type levels without zinc. Lane 1, D39 *rpsL1* Δ*cps* (IU1824); lane 2, D39 *rpsL1* Δ*cps ftsX*(Q111A L115C)/*bgaA*::*tet-*P_Zn_*-ftsX^+^* (IU13064); lane 3, D39 *rpsL1* Δ*cps ftsX*(L115A M119A)/*bgaA*::*tet-*P_Zn_*-ftsX^+^* (IU13066); lane 4, D39 *rpsL1* Δ*cps* Δ*ftsX*/*bgaA*::*tet-*P_Zn_*-ftsX^+^*. (C) FtsX(L115A) and FtsX(M119A) are expressed at nearly wild-type levels without zinc. Lane 1, D39 *rpsL1* Δ*cps* (IU1824); lane 2, D39 *rpsL1* Δ*cps ftsX*(L115A)/*bgaA*::*tet-*P_Zn_*-ftsX^+^* (IU12521); lane 3, D39 *rpsL1* Δ*cps ftsX*(M119A)/*bgaA*::*tet-*P_Zn_*-ftsX^+^* (IU12637); lane 4, D39 *rpsL1* Δ*cps* Δ*ftsX*/*bgaA*::*tet-*P_Zn_*-ftsX^+^* (IU13461). (D) FtsX(E109Q) and FtsX(N131D) are expressed at nearly wild-type levels without zinc. Lane 1, D39 *rpsL1* Δ*cps* (IU1824); lane 2, D39 *rpsL1* Δ*cps ftsX*(E109Q)/*bgaA*::*tet-*P_Zn_*-ftsX^+^* (IU13088); lane 3, D39 *rpsL1* Δ*cps ftsX*(N131D)/*bgaA*::*tet-*P_Zn_*-ftsX^+^* (IU13089); lane 4, D39 *rpsL1* Δ*cps* Δ*ftsX*/*bgaA*::*tet-*P_Zn_*-ftsX^+^* (IU13461). (E) FtsX with (Gly_3_Ser)_2_ after residue 51 is expressed, whereas FtsX with (Gly_3_Ser)_2_ after residue 173 is not expressed. These are referred to as *ftsX* N-term ECL1(Gly_3_Ser)_2_ and *ftsX* C-term ECL1(Gly_3_Ser)_2_, respectively. Lane 1, D39 *rpsL1* Δ*cps* (IU1824); lane 2, D39 *rpsL1* Δ*cps ftsX* N-term ECL1(Gly_3_Ser)_2_/*bgaA*::*tet-*P_Zn_*-ftsX^+^* (IU12629); lane 3, D39 *rpsL1* Δ*cps ftsX* C-term ECL1(Gly_3_Ser)_2_/*bgaA*::*tet-*P_Zn_*-ftsX^+^* (IU12869); lane 4, D39 *rpsL1* Δ*cps* Δ*ftsX*/*bgaA*::*tet-*P_Zn_*-ftsX^+^* (IU13461). These experiments were performed two to three times independently.

We next constructed three classes of amino acid substitution or insertion mutants (Table 4) in an effort to disrupt the FtsX_ECL1_–PscB_CC_ interaction defined by NMR spectroscopy. These are designated class I (single amino acid changes), class II (multiple amino acid changes), and class III [insertion of a (Gly_3_Ser)_2_ linker] mutants. Class I strains were made by introducing single-amino-acid substitutions in the merodiploid strain and measuring growth or morphology defects (Table 4, Fig. S8). Class I mutants targeted both the α2 helix and the loop region (residues 107 to 120 and 121 to 130, respectively) of the FtsX_ECL1_ helical lobe (Table 4, Fig. 3A, Fig. S8A). Single-amino-acid-substitution mutations of FtsX (including E109A [a change of E to A at position 109], L115A, M119A, W123A, F126A, N131A, and N131D) resulted in morphological defects without Zn-Mn (Table 4, Fig. S8C and D) but did not induce a measurable growth phenotype (Table 4, Fig. S8B). The expression of FtsX(L115A) resulted in cell shape defects (aspect ratio, length, width, and volume) (Fig. S8C and D), while the expression of FtsX(M119A) only resulted in a change in cell volume (Fig. S8D). These differences were not due to misexpression of FtsX, as Western blot analysis indicates that all mutant proteins were expressed at or near wild-type levels (Fig. 4C). Two other single-amino-acid substitutions (E109Q and Q111A) did not strongly affect growth, morphology, or expression (Table 4, Fig. 4D).

**TABLE 4:** Amino acid changes made *in vivo* to disrupt the FtsX_ECL1_-PcsB interaction^*a*^

Category of change(s) made^*b*^	Amino acid change(s) or location of insertion	Defect(s) in shape and/or growth	Location(s) in FtsX_ECL1_
Class I	E109A	Shape only	α2 helix
	E109Q	No	α2 helix
	Q111A	No^*c*^	α2 helix
	L115A	Shape only	α2 helix
	M119A	Shape only	α2 helix
	W123A	Shape only	Loop^*d*^
	F126A	Shape only	Loop^*d*^
	N131A	Shape only	Loop^*d*^
	N131D	Shape only	Loop^*d*^

Class II	E109A N131A	Shape only	α2 helix, loop^*d*^
	Q111A L115C	Shape only	α2 helix
	L115A M119A	Yes	α2 helix
	E109A Q111A L115A M119A	Yes	α2 helix
	F126A W123A N131A	Yes	Loop^*d*^

Class III^*e*^	Residue 51	Yes	Post-TM1
	Residue 78	Shape only	β-Hairpin
	Residue 173	NA^*f*^	Pre-TM2

a See the supplemental material for the corresponding strain number and strain construction for each amino acid change or insertion.

b Class I, single amino acid changes; class II, multiple amino acid changes; class III, insertion of a (Gly_3_Ser)_2_ linker.

c In the absence of zinc, this strain had no morphology defects. Morphology defects were observed at 3 h when the strain was expressing both the wild-type copy of FtsX and FtsX(Q111A).

d Refers to the unstructured loop after the α2 helix in the helical lobe of the FtsX_ECL1_ structure.

e These strains have an insertion of the amino acid sequence GGGSGGGS after the indicated residue.

f NA, not applicable, since the strain did not express FtsX with the (Gly_3_Ser)_2_ insertion. See Fig. 4E for Western blot data.

10.1128/mBio.02622-18.8 FIG S8 Expression of FtsX(L115A) or FtsX(M119A) results in mild morphological defects. (A) Amino acid changes made are mapped to the structure of FtsX_ECL1_. The α carbon of each residue is shown as a colored sphere. The orange-to-red coloring on the FtsX_ECL1_ structure represents the peak height changes in the ^1^H-^15^N HSQC spectra upon the addition of 2 molar equivalents of PscB_CC_ to FtsX_ECL1_. (B) Representative growth curves of strains expressing FtsX(L115A) or FtsX(M119A) versus FtsX depletion and wild-type strains. These strains were grown with or without 0.45 mM ZnCl_2_ supplemented with 0.045 mM MnSO_4_ (indicated as +Zn). Strains shown are as follows: black circle, strain D39 *rpsL1* Δ*cps* wild-type parent (1, strain IU1824); grey circle, IU1824 +Zn; dark blue square, D39 *rpsL1* Δ*cps* Δ*ftsX*::P-*aad9*/*bgaA*::*tet-*P_Zn_*-ftsX^+^* (2, strain IU12376); light blue square, IU12376 +Zn; red triangle, D39 *rpsL1* Δ*cps ftsX*(L115A)/*bgaA*::*tet-*P_Zn_*-ftsX^+ ^*(3, strain IU12521); pink triangle, IU12521 +Zn; brown inverted triangle, D39 *rpsL1* Δ*cps ftsX*(M119A)/*bgaA*::*tet-*P_Zn_*-ftsX^+ ^*(4, strain IU12637); light brown inverted triangle, IU12637 +Zn. This experiment was performed three times with similar results. (C) Representative images of strains at 6 hours growth. The genotype of the strain shown is indicated above each panel. No Zn or +Zn indicates whether or not Zn-Mn was added. %, percentage of cells in the population that are morphologically similar to the images shown. More than 50 cells per strain per experimental repeat were analyzed. These experiments were performed three times independently with similar results. Scale bar is equal to 1 µm. (D) Lengths, widths, aspect ratios, and relative cell volumes of strains at 6 h of growth. Strains are indicated according to the numbering in panel B. More than 50 cells were measured per strain and condition over two experimental replicates. For statistical analysis, the Kruskal-Wallis test (one-way ANOVA) with Dunn’s multiple comparison posttest was used to determine if lengths, widths, aspect ratios, and relative cell volumes were significantly different between strains and conditions. ns, not significant; *, *P* < 0.05; **, *P* < 0.005; ***, *P* < 0.0005. Download FIG S8, EPS file, 5.8 MB. Copyright © 2019 Rued et al. 2019 Rued et al. This content is distributed under the terms of the Creative Commons Attribution 4.0 International license.

In contrast to the somewhat modest physiological impact of class I substitutions, selected class II mutants (Table 4) exhibited severe morphological and growth defects (Fig. 3, Table 4, Fig. S9). In strains with mutations targeting the α2 helix [FtsX(E109A Q111A L115A M119A)] or the coil [FtsX(F126A W123A N131A)], ≥98% of cells had severe growth and morphology defects. The growth and morphology of these strains were similar to those of cells in which *ftsX* was depleted (Fig. 3B to D). Cells expressing these *ftsX* alleles became significantly rounder and shorter (Fig. 3D) and showed growth inhibition in the absence of zinc (Fig. 3B). Importantly, in the presence of zinc, the cells were indistinguishable from wild-type cells at 3 h (Fig. 3D). At 6 h with zinc, these cells exhibited changes in width and volume, which could be due to overexpression of FtsX at this time point or the expression of wild-type and mutant FtsX simultaneously (Fig. 3D). Western blotting confirmed that FtsX(E109A Q111A L115A M119A) and FtsX(F126A W123A N131A) were expressed in the absence of zinc at 6 h postdepletion (Fig. 4A). The triple FtsX(F126A W123A N131A) mutant expressed in the absence of zinc migrated slightly higher than wild-type FtsX on an SDS-PAGE gel, but it was expressed (Fig. 4A). Taken together, these results reveal that both the α2 helix and the loop in the helical lobe of FtsX_ECL1_ are important for FtsX function *in vivo* and confirm the functional importance of the physical interaction of FtsX and PcsB mapped by NMR spectroscopy.

10.1128/mBio.02622-18.9 FIG S9 Cells expressing FtsX(L115A M119A) have severe defects in morphology and growth. Cells expressing FtsX(Q111A L115C) have defects in morphology but not growth. (A) Amino acid changes made mapped to the structure of FtsX_ECL1_. The α carbon of each residue is shown as a colored sphere. The orange-to-red coloring on the FtsX_ECL1_ structure represents the peak height changes in the ^1^H-^15^N HSQC spectra upon the addition of 2 molar equivalents of PscB_CC_ to FtsX_ECL1_. (B) Representative growth curves of strains expressing FtsX(Q111A L115C) and FtsX(L115A M119A) versus FtsX depletion and wild-type strains. These strains were grown with or without 0.45 mM ZnCl_2_ and 0.045 mM MnSO_4_ (indicated as +Zn). Strains shown are as follows: black circle, strain D39 *rpsL1* Δ*cps* wild-type parent (1, strain IU1824); grey circle, IU1824 +Zn; blue square, D39 *rpsL1* Δ*cps* Δ*ftsX*::P-*aad9*/*bgaA*::*tet-*P_Zn_*-ftsX^+^* (2, strain IU12376); light blue square, IU12376 +Zn; green inverted triangle, D39 *rpsL1* Δ*cps ftsX*(Q111A L115C)/*bgaA*::*tet-*P_Zn_*-ftsX^+ ^*(3, strain IU13064); light green inverted triangle, IU13064 +Zn; dark purple diamond, D39 *rpsL1* Δ*cps ftsX*(L115A M119A)/*bgaA*::*tet-*P_Zn_*-ftsX^+^* (4, strain IU3066); light purple diamond, IU13066 +Zn. This growth curve experiment was repeated three times with similar results. (C) Representative images of strains at 6 h of growth. The genotype of the strain shown is indicated above each panel. No Zn or +Zn indicates whether or not Zn-Mn was added. %, percentage of cells in the population that are morphologically similar to the images shown. More than 50 cells were counted per experimental run, per condition and strain. These experiments were performed three times independently with similar results. Scale bar is equal to 1 µm. (D) Length, width, aspect ratio, and relative cell volume of strains at 6 hours growth. Strains are indicated according to the numbering in panel B. Greater than 50 cells were measured per strain and condition over two experimental replicates. For statistical analysis, a Kruskal-Wallis test (one-way ANOVA) with Dunn’s multiple comparison posttest was used to determine if length, width, aspect ratio, and relative cell volume were significantly different between strains and conditions. ns, not significant; *, *P* < 0.05; **, *P* < 0.005; ***, *P* < 0.0005. Download FIG S9, EPS file, 5.9 MB. Copyright © 2019 Rued et al. 2019 Rued et al. This content is distributed under the terms of the Creative Commons Attribution 4.0 International license.

Some of the class II mutants we characterized had just two amino acid changes in the α2 helix or the extended loop of the helical lobe (Table 4, Fig. S9). We observed that 99% of cells expressing FtsX(L115A M119A) exhibited strong growth and morphology defects at 6 h in the absence of zinc (Table 4, Fig. S9B and C), and these cells displayed decreases in length, width, and volume relative to those of wild-type cells (Fig. S9D). This mutant was expressed at nearly wild-type levels in the absence of zinc (Fig. 4B). These data confirm that the tandem L115A M119A substitution disrupts FtsX function, even though these substitions result in only slight morphological defects as individual single mutations (Table 4, Fig. S8B to D, Fig. S9B to D). Another double mutant, FtsX(Q111A L115C), induced the formation of long chains and a “boxy” cell morphology (Table 4, Fig. S9C). This mutant resulted in shorter cells with a significantly different aspect ratio than the wild type (Fig. S9D), but this strain had no growth phenotype (Fig. S9B). In contrast, another double substitution class II mutant, FtsX(E109A N131A) (Table 4), exhibited no strong morphology or growth defects.

Finally, class III insertion mutants (Table 4) were constructed and used to evaluate whether other regions of FtsX_ECL1_ were important for the FtsX_ECL1_-PcsB interaction or for FtsX function. We inserted a (Gly_3_Ser)_2_ flexible linker either approximately where FtsX_ECL1_ is predicted to enter (residue 51) or exit (residue 173) the membrane bilayer or in the β-hairpin, which exhibits significant conformational disorder over a range of timescales (Fig. 1A). An insertion after residue 51 in FtsX_ECL1_ was detrimental to both growth and morphology (Table 4), and this insertion did not disrupt FtsX expression (Fig. 4E). The insertion after residue 173 in FtsX_ECL1_ also caused growth and morphology defects, but this FtsX variant was not expressed in cells (Table 4, Fig. 4E). The β-hairpin (Gly_3_Ser)_2_ insertion (Fig. 1B, see appendix in Text S1) was introduced after amino acid 78 of FtsX_ECL1_, which corresponds to the tip of the β-turn in the β-hairpin (Fig. 1B). This strain also exhibited no growth defect, but these cells were significantly smaller, although only at the 3 hr time point (see appendix in Text S1). We conclude that the β-hairpin does not play a major role in FtsX-PcsB interaction, consistent with the NMR mapping experiments.

### FtsX_ECL1_(L115A M119A) is stably folded and unable to bind PscB_CC_.

We reasoned that if the defects observed in class I and class II mutants were due to the disruption of the FtsX_ECL1_–PscB_CC_ interaction, this should affect the affinity of this interaction as measured by ITC. We first characterized the L115A M119A double mutant by ^1^H-^15^N NMR and CD spectroscopy to confirm its structural integrity. The CD spectrum resembled that of the wild type, as did the ^1^H-^15^N HSQC spectrum, with clear chemical shift perturbations only among those resonances in the immediate vicinity of the double substitution (Fig. S10B). Both pieces of data suggest a local rather than global perturbation of the α2-loop lobe in the FtsX_ECL1_ structure upon introduction of the L115A M119A double substitution. As anticipated, titration of PscB_CC_(47–267) with FtsX_ECL1_(L115A M119A) reveals no detectable binding (no observable heat) (Fig. 2B, Table 3) compared to the results using wild-type FtsX_ECL1_. These data confirm that the helical lobe of FtsX_ECL1_ interacts with PscB_CC_ and that this interaction is required for viability and proper cell shape.

10.1128/mBio.02622-18.10 FIG S10 ^1^H-^15^N HSQC spectra of ^15^N-labeled FtsX_ECL1_(E109A Q111A L115A M119A), FtsX_ECL1_(L115A M119A), and FtsX_ECL1_(W123A F126A N131A) show perturbation of residues in the lower lobe of FtsX_ECL1_. (A) ^1^H-^15^N HSQC spectrum of 50 µM ^15^N-labeled FtsX_ECL1_(E109A Q111A L115A M119A) compared to the wild-type FtsX_ECL1_ spectrum. Wild-type FtsX_ECL1_ peaks are indicated in black, and FtsX_ECL1_(E109A Q111A L115A M119A) peaks are indicated in light blue. Below the spectra, peaks that are no longer in the area of the original wild-type peak are indicated by their α carbon as a sphere on the FtsX_ECL1_ structure. Overlapping peaks are indicated in white. (B) ^1^H-^15^N HSQC spectrum of 50 µM ^15^N-labeled FtsX_ECL1_(L115A M119A) compared to the wild-type FtsX_ECL1_ spectrum. Wild-type FtsX_ECL1_ peaks are indicated in black, and FtsX_ECL1_(L115A M119A) peaks are indicated in green. Below the spectra, peaks that are no longer in the area of the original wild-type peak are indicated by their α carbon as a sphere on the FtsX_ECL1_ structure. Overlapping peaks are indicated in white. (C) ^1^H-^15^N HSQC spectrum of 50 µM ^15^N-labeled FtsX_ECL1_(W123A F126A N131A) compared to the wild-type FtsX_ECL1_ spectrum. Wild-type FtsX_ECL1_ peaks are indicated in black, and FtsX_ECL1_(W123A F126A N131A) peaks are indicated in purple. Below the spectra, peaks that are no longer in the area of the original wild-type peak are indicated by their α carbon as a sphere on the FtsX_ECL1_ structure. Overlapping peaks are indicated in white. (D) Circular dichroism (CD) spectroscopy results for wild-type FtsX_ECL1_, FtsX_ECL1_(L115A M119A), FtsX_ECL1_(E109A Q111A L115A M119A), and FtsX_ECL1_(W123A F126A N131A) plotted as the function of mean residue ellipticity (deg · cm^2^ dmol^−1^) × 10^3^ versus wavelength (nm). Download FIG S10, EPS file, 19.2 MB. Copyright © 2019 Rued et al. 2019 Rued et al. This content is distributed under the terms of the Creative Commons Attribution 4.0 International license.

In contrast to the L115A M119A double mutant, the severely functionally compromised representative triple mutant FtsX_ECL1_(W123A F126A N131A) and quadruple mutant FtsX_ECL1_(E109A Q111A L115A M119A) exhibited more pronounced structural perturbations that nonetheless map only to the helical lobe. The triple mutant was indistinguishable from the L115A M119A and wild-type FtsX_ECL1_ derivatives by CD spectroscopy, while the quadruple mutant exhibited less molar ellipticity, or secondary structure (Fig. S10D). Inspection of their ^1^H-^15^N HSQC spectra reveals that although the core and β-hairpin domains essentially resemble those of the wild type, each of these mutants exhibits considerable perturbation of resonances throughout the helical lobe (Fig. S10A and C). Since these mutants are functionally compromised, these structural findings strongly support the conclusion that the structural integrity of the lower lobe is essential for the physical interaction with PcsB and the function of FtsX in pneumococcal cells.

## DISCUSSION

This study presents a comprehensive analysis of the solution and X-ray structures of the outward-facing large extracellular loop of FtsX (FtsX_ECL1_) from S. pneumoniae and defines a physical interaction site with the coiled-coil domain of peptidoglycan hydrolase PcsB (PscB_CC_). Our FtsX_ECL1_ structures reveal a globular fold that, while similar to the large extracellular loop of FtsX from M. tuberculosis (16), is characterized by unique features. The upper β-hairpin distinguishes S. pneumoniae FtsX_ECL1_ from that of M. tuberculosis, and despite being characterized by significant conformational dynamics on a range of timescales, it is not required for the interaction of S. pneumoniae FtsX_ECL1_ with PcsB. The function of this domain is not well defined by our data, but it could play a role in another process, e.g., FtsX dimerization, interaction with the small extracellular loop (ECL2), or interaction with another domain of PcsB. Alternatively, it could be a result of the difference in cell wall architecture between S. pneumoniae and M. tuberculosis. On the other hand, the helical lobe of FtsX_ECL1_, common to the FtsX structures of both S. pneumoniae and M. tuberculosis, is vital for the interaction with PcsB *in vitro*, and this interface is functionally important *in vivo*. Increasing numbers of Ala substitutions tested here increasingly disrupt this interaction and ultimately cause dramatic growth and morphology defects, indicating that the helical lobe of FtsX_ECL1_ is essential for regulation of PcsB during cell division. Interestingly, this region of FtsX_ECL1_ corresponds with the region shown to be important for the interaction of M. tuberculosis FtsX_ECD_ with its PG hydrolase, RipC (16). This suggests that the helical lobe could be a conserved functional determinant for the interaction of FtsX with cognate hydrolases or adaptor proteins across many species of bacteria.

**FIG 5:**
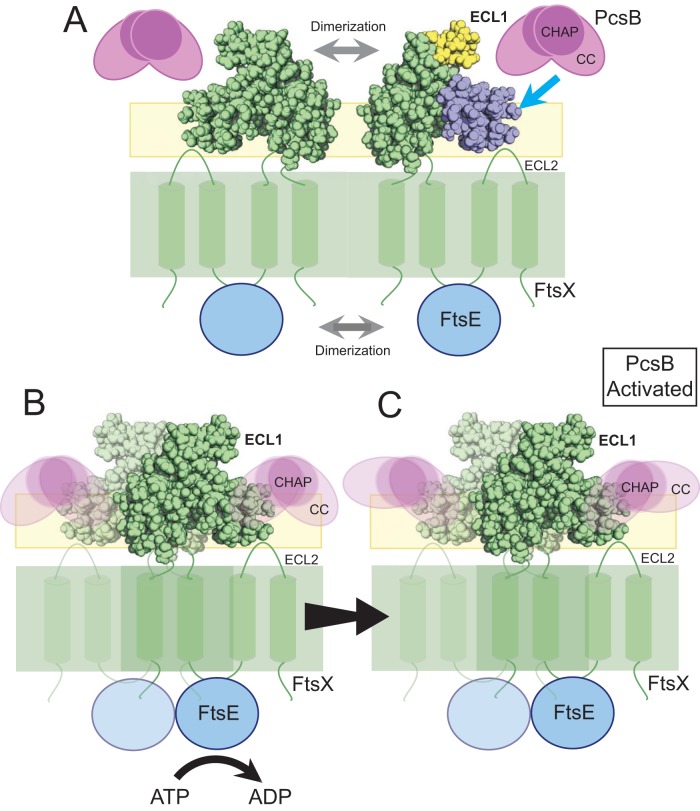
Model for the activation of PcsB by FtsX_ECL1_. (A) FtsEX dimerizes to form the active complex. PcsB is secreted into the extracellular milieu. Attraction of PcsB to the area of active FtsX complexes might be mediated by its propensity to interact with membranes (32). The ECL1 and ECL2 loops are indicated on FtsX. FtsX_ECL1_ is shown in green, with the β-hairpin and α-helical lobe shaded in yellow and blue, respectively. (B) After formation of the active complex, ATP hydrolysis by FtsE causes a conformational change in FtsX. (C) PcsB interacts with FtsX_ECL1_ via its coiled-coil domain, and this interaction causes activation of the peptidoglycan hydrolytic activity of PcsB. PcsB, along with other factors in the cell, allows cell division to proceed normally. Functional FtsX, FtsE, and PcsB are all required for efficient cell division.

We propose that the helical lobe of FtsX_ECL1_ is important for the activation of cognate hydrolase activity either directly or indirectly through adaptors (Fig. 5). The exact role of the second extracellular loop of FtsX (FtsX_ECL2_) is unknown, but it may also regulate this process, as temperature-sensitive mutations in *pcsB* were found to be suppressed by mutations in the coding region for FtsX_ECL2_ (20). Previous work suggests that FtsEX forms a dimer (32), as dimerization of the FtsE ATPase domain is likely a necessary condition for ATP hydrolysis (Fig. 5A) (36, 37). After formation of the complex, ATP hydrolysis by FtsE results in a conformational change in FtsX, releasing PcsB from what we anticipate is an inhibited state (Fig. 5B and C) (22). This interaction is mediated by the helical lobe of FtsX_ECL1_, although the membrane and, possibly, lipid binding by FtsX_ECL1_ and FtsX_ECL2_ itself may also play a role. We propose that the interaction of FtsX_ECL1_ with the PcsB coiled-coil domain communicates release of the PcsB CHAP domain from an inhibited state and, thus, is important for modulating PG hydrolysis by PcsB (Fig. 5B and C).

Recently, the structure of Aggregatibacter actinomycetemcomitans MacB was reported and suggested to be a structural paradigm for the ABC3 transporter superfamily that includes FtsX (38). MacB was proposed to function as a mechanical pump to drive enterotoxin transport through TolC in E. coli (38). Crow et. al found that MacB itself did not transport enterotoxin but drove TolC to transport it instead, due to the lack of a central cavity in the MacB structure (38). As such, they proposed MacB as a model for other so-called “mechanotransmitters” belonging to this same ABC3 transporter superfamily. While this proposed function of mechanotransmission may well characterize MacB and FtsX, the overall structure of FtsX_ECL1_ from S. pneumoniae is clearly distinct from the periplasmic domain of MacB, thus revealing that MacB does not readily provide a structural basis for understanding FtsX-dependent peptidoglycan hydrolases. Future work using reconstituted FtsEX and PcsB complexes will allow for understanding how this common mechanotransmission principle extends throughout the ABC3 superfamily.

## MATERIALS AND METHODS

### NMR spectroscopy.

Spectra of ^15^N- or ^15^N^13^C-labeled FtsX_ECL1_ were recorded at 298 K on Varian (Agilent) DDR 600- or 800-MHz spectrometers equipped with cryogenic probes in the METACyt Biomolecular NMR Laboratory at Indiana University Bloomington. NMR samples contained 50 mM potassium phosphate, pH 7.0, 50 mM NaCl, and 10% (vol/vol) D_2_O, with 0.2 mM 4,4-dimethyl-4-silapentane-1-sulfonic acid (DSS) for chemical shift referencing. The typical concentrations of FtsX_ECL1_ were 50 µM for ^15^N HSQC spectra and 400 µM for triple-resonance and dynamics experiments. ^1^*J*_HN_ splittings for residual dipolar couplings (RDCs) were measured using two-dimensional in-phase/anti-phase (2D IPAP) [^15^N, ^1^H]-HSQC spectra (39), recorded on an isotropic sample and on a sample aligned with 20 mg/ml phage *Pf*1 (ASLA Biotech). ^1^*D*_HN_ was calculated from ^1^*D*_HN_ = ^1^*J*_HN_ (anisotropic) – ^1^*J*_HN_ (isotropic). Aromatic sidechains were assigned using the HBCBCGCDHD and HBCBCGCDCEHE experiments (40). For experiments detecting PscB_CC_ binding, ^15^N FtsX_ECL1_ was kept at 50 µM, and ^1^H-^15^N HSQC spectra were recorded with the following concentrations of PscB_CC_(47–267): 0, 50 µM, 100 µM, 200 µM, and 400 µM. nmrPipe, Sparky, CARA (http://cara.nmr.ch), CCPNMR, and NMRbox (41–44) were used for data processing and analysis. Resonance assignments and dynamics data are available in the BMRB under accession code 30523. These NMR data were used to calculate and refine the solution structure of ECL1 (see supplemental materials and methods in Text S1), with the ensemble of the 20 lowest-energy structures (see Table 1 for structure statistics) deposited in the Protein Data Bank (PDB code 6MK7).

### X-ray crystallography.

Crystallization screenings were performed by using high-throughput crystallization techniques in a NanoDrop robot with Innovadyne SD-2 microplates (Innovadyne Technologies, Inc.) and screening using PACT Suite and JCSG Suite (Qiagen), JBScreen Classic 1 to 4 and 6 (Jena Bioscience), and Crystal Screen, Crystal Screen 2, and Index HT (Hampton Research). Positive conditions in which crystals grew were optimized by the sitting-drop vapor-diffusion method at 291 K by mixing 1 µl of protein solution and 1 µl of precipitant solution, equilibrated against 150 µl of precipitant solution in the reservoir chamber. The best crystals were obtained under a crystallization condition of a solution containing 0.1 M sodium citrate, pH 5.6, 0.2 M potassium-sodium tartrate, and 2 M ammonium sulfate. These crystals were further optimized by cocrystallization in the presence of detergents dodecyltrimethylammonium chloride (detergent 1 [D1]; 46 µM), *n*-undecyl-β-d-maltoside (detergent 2 [D2]; 0.59 mM), and *n*-decyl-β-d-maltoside (detergent 3 [D3]; 1.8 mM). Crystals were cryoprotected in the precipitant solution supplemented with 25% (vol/vol) glycerol prior to flash cooling at 100 K. Diffraction data were collected in the XALOC beamline at the ALBA synchrotron (CELLS-ALBA, Spain), using a Pilatus 6 M detector and a wavelength of 0.979257 Å. Crystals diffracted up to 2.0- to 2.3-Å resolution and belonged to the P 4_3_ 2_1_ 2 space group. The collected data sets were processed with XDS (45) and Aimless (46). Two FtsX_ECL1_ molecules were found in the asymmetric unit for all three structures, yielding Matthews coefficients of 2.59 Å^3^/Da (FtsX_ECL_-D1), 3.49 Å^3^/Da (FtsX_ECL_-D2), and 2.49 Å^3^/Da (FtsX_ECL_-D1) (47) and solvent contents of 52.5% (FtsX_ECL_-D1), 64.7% (FtsX_ECL_-D2), and 50.7% (FtsX_ECL_-D3). Structure determination was performed by molecular replacement using the online server Morda (http://www.biomexsolutions.co.uk/morda/). Refinement and manual model building were performed with Phenix (48) and Coot (49), respectively. The data for FtsX_ECL_-D1 and FtsX_ECL_-D3 presented translational noncrystallographic symmetry (fractional coordinates of [−0.498, −0.498, 0.50] and height relative to origin of 79.60% for FtsX_ECL_-D1, and fractional coordinates of [−0.498, −0.498, 0.50] and height relative to origin of 78.70% for FtsX_ECL_-D3) that were treated with Phenix (48). The stereochemistry of the final model was checked by MolProbity (50). Data collection and processing statistics are shown in Table 2.

### Bacterial strains, plasmids, and growth conditions.

Bacterial strains and plasmids used in this study are listed in the supplemental material. S. pneumoniae strains were derived from IU1945, an unencapsulated derivative of serotype 2 S. pneumoniae strain D39 (51). Strains were grown on Trypticase soy agar II with 5% (vol/vol) defibrinated sheep blood (TSAII-BA) plates or in Becton, Dickinson brain heart infusion (BHI) broth at 37°C in an atmosphere of 5% CO_2_. E. coli strains for protein expression were derived from strain BL21(DE3) (catalog number C2527H; NEB). E. coli strains were grown in Luria-Bertani (LB) broth or in M9 minimal medium supplemented with ^15^NH_4_Cl at 37°C with shaking at 150 rpm. When required, tetracycline (0.25 to 2.5 µg/ml), kanamycin (250 µg/ml), spectinomycin (150 µg/ml), streptomycin (250 µg/ml), ampicillin (100 µg/ml), and/or isopropyl β-d-1-thiogalactopyranoside (IPTG) (1 mM) was added to S. pneumoniae or E. coli culture medium. S. pneumoniae strains requiring zinc for expression of essential genes were grown with 0.45 mM ZnCl_2_ and 0.045 MnSO_4_.

### Growth curve experiments and phase-contrast microscopy of strains.

For physiological and morphological analyses of strains, cells were inoculated from frozen glycerol stocks into BHI broth, serially diluted, and incubated for 10 to 12 h statically at 37°C in 5% CO_2_ overnight. If zinc was required for growth of cultures, 0.45 mM ZnCl_2_ and 0.045 MnSO_4_ were added to overnight tubes. The next day, cultures ranging from an optical density at 620 nm (OD_620_) of ≈0.05 to 0.4 were diluted into fresh BHI to an OD_620_ of ≈0.003 in 4-ml volumes, and two identical cultures for each strain were prepared, one with 0.45 mM ZnCl_2_–0.045 MnSO_4_ and one without. These cultures were grown under the same growth conditions as described above. Growth was monitored turbidimetrically every 45 min to 1 h with a Genesys 2 spectrophotometer (Thermo Scientific). For microscopic analyses, samples (1 to 2 μl) were taken at 3 h and 6 h and examined using a Nikon E-400 epifluorescence phase-contrast microscope with a 100× Nikon Plan Apo oil-immersion objective (numerical aperture, 1.40) connected to a CoolSNAP HQ2 charge-coupled device (CCD) camera (Photometrics). Images were processed using NIS-Elements AR software (Nikon), and measurements and calculation of cell width, length, volume, and aspect ratio were performed as described previously (52, 53). Statistical significance was determined using GraphPad Prism (GraphPad Software, Inc.) by comparing values for cell width, length, volume, and aspect ratio measured for at least 50 cells over two experimental replicates. To determine if values were significantly different between strains and conditions, the Kruskal-Wallis test (one-way analysis of variance [ANOVA]) with Dunn’s multiple comparison posttest was used.

For additional materials and methods, please see Text S1.

### Data availability.

The ensemble of the 20 lowest-energy structures from using NMR data to calculate and refine the solution structure of ECL1 has been deposited in the Protein Data Bank (PDB code 6MK7). The atomic coordinates of FtsX_ECL1_ determined by cocrystallization in the presence of detergents 1, 2, and 3 have been deposited in the Protein Data Bank with PDB codes 6HE6, 6HEE, and 6HFX, respectively. NMR spectroscopy resonance assignments and dynamics data are available in the BMRB under accession code 30523.
