# Supplementary material for: Structure of the Large Extracellular Loop of FtsX and Its Interaction with the Essential Peptidoglycan Hydrolase PcsB in Streptococcus pneumoniae
Source: mBio. 2019 Jan 29;10(1):e02622-18. doi: 10.1128/mBio.02622-18 (PMC6355983; doi:10.1128/mBio.02622-18)

SUPPLEMENTAL MATERIALS AND METHODS

Construction of *E. coli* strains with protein expression plasmids. Transformation of *E. coli* α-select gold efficiency (Bioline, Bio-85027) or BL21(DE3) (NEB, C2527H) to obtain derivative strains was performed by transformation of plasmids as outlined by supplier manuals. Plasmids were derived for transformation as follows. Linear DNA amplicons with the insert of choice and the *EcoR1* and *NcoI* digest sites on the 5' and 3' end, respectively, were obtained by PCR from D39 genomic DNA. All primers used in these reactions are listed in Table S1. pHis-parallel plasmid (1) was obtained from strain IU6814 (Table S1) using the Qiaprep Spin Miniprep Kit per manufacturer's instructions (Qiagen, 27106). pHis plasmid and inserts were digested with *EcoR1* and *NcoI* for at least 1 hour at 37°C, and then were incubated at 65°C for 20 minutes to inactivate restriction enzymes. Digested pHis plasmids and inserts were then ligated together using T4 DNA ligase (NEB, M0202S) at room temperature for 2 hours. Transformation of ligated plasmids was then performed as specified by manufacturer's instructions for *E. coli* α-select gold efficiency (Bioline, Bio-85027) or BL21(DE3) (NEB, C2527H).

Expression and purification of ^{15}N- or unlabeled FtsX$_{ECL1}$ and FtsX$_{ECL1}$ with amino acid changes for NMR. Plasmids for expression of FtsX$_{ECL1}$ (FtsX amino acids 47-168, 119 residues) and its derivatives are listed in Table S1. ^{15}N-labeled and unlabeled proteins were purified as previously described (2), with the following changes. Instead of sonication, cultures expressing FtsX$_{ECL1}$ were lysed by passing pellets suspended in lysis buffer (25 mM Tris-HCl, pH 8, 200 mM NaCl, 10 mM imidazole) through a French Press (ThermoSpectronic French Pressure Cell Press) at 18,000 psi. Lysed cells were

then spun for 90 min at 12,000*xg*, 4°C. For the production of unlabeled protein, cultures were grown and induced in LB broth.

$FtsX_{ECL1}$ NMR Solution Structure Refinement Initial structure calculations with automated assignment of ^{15}N-edited and aliphatic and aromatic ^{13}C-edited NOESY spectra performed using CYANA 3.97, incorporating RDC restraints (3). The structure was further refined with manual NOE assignment using XPLOR-NIH. The solution structure builds upon the previously published backbone and sidechain assignment deposited in the BioMagResBank, under the accession number 26534 (2), and was calculated using 1713 Nuclear Overhauser Effect (NOE) distance restraints, including 615 long-range NOEs, as well as 110 chemical shift-derived backbone dihedral angle restraints (4). However, the protruding nature of the helical lobe and the β-hairpin led to an inadequate network of NOEs to adequately define the orientation of these two subdomains, requiring the use of Residual Dipolar Coupling (RDC) restraints. The 121 measured $^{1}D_{HN}$ RDC values obtained by weak alignment in Pf1 filamentous phage correspond well to previously determined secondary structure elements (2). Inclusion of RDC restraints in refinement of the solution structure led to a strong correlation between experimentally measured RDCs and predicted RDCs back calculated from the structural ensemble using the software PALES (5). The correlation between experimental RDCs and fitted values using the crystallographic structure is also strong, indicating that the crystal structure is an accurate representation of the solution conformation. However, correlation by residues indicates the experimental RDCs match poorly in the β-hairpin and region between α2 and β5. The motions cause the experimental values to average out to 0, which is not accounted for in the fitting to static structures. The ensemble of 20

lowest energy structures have been deposited in the Protein Data Bank (accession code 6MK7).

Cloning, expression and purification of FtsX$_{ECL1}$ for crystallization. FtsX$_{ECL1}$ from *S. pneumoniae* D39 (residues 49-166, 117 residues) was purified by using the tandem affinity CHiC-tag as previously described by (6). In brief, *FtsX$_{ECL1}$* fused to the CHiC-tag by overlap extension PCR was cloned into the expression vector pRSET A (Invitrogen). *E. coli* BL21 (DE3) (Invitrogen) cells containing the plasmid were grown at 37°C with shaking. When reaching OD$_{600}$ ~0.5, expression of CHiC-fused FtsX$_{ECL1}$ was induced by adding 0.1 mM isopropyl-β-D-thiogalactopyranoside (IPTG). After a 3 hr incubation at 28° C cells from 2 L of cell culture were harvested at 5,000*xg* and resuspended in 40 ml 10 mM Tris-HCl (pH 7.4) containing 100 mM NaCl. The cells were lysed by adding lysozyme to a final concentration of 1 mg/ml (Sigma Aldrich). CHiC-FtsX$_{ECL1}$ was purified from the soluble protein fraction by DEAE-cellulose affinity chromatography as described by (7). The CHiC-tag was then separated from FtsX$_{ECL1}$ by digestion with TEV Protease (Invitrogen) at 30°C for 3 h. The free CHiC-tag, undigested CHiC-FtsX$_{ECL1}$ and TEV Protease, all of which contain a 6xHis-tag, were removed by Ni^{2+}-NTA affinity chromatography. The flow-through which contained FtsX$_{ECL1}$ was concentrated using an Amicon Ultra 15 mL Centrifugal Filter (3 kDa molecular weight cut-off) to a final concentration of 15 mg/ml in 10 mM Tris-HCl (pH 7.4) and 50 mM NaCl.

Expression and purification of ^{15}N or unlabeled PcsB coiled coil domain. Plasmids for expression of PcsB coiled coil domain (PcsB-CC, amino acids 47-267 or PcsB-CC, amino acids 47-254) and are listed in Table S1. PcsB-CC (47-267) and PcsB-CC (47-

254) were induced and grown in the same manner as unlabeled and ^{15}N labeled $FtsX_{ECL1}$. Cell pellets obtained from growth and induction were resuspended in lysis buffer (25 mM Tris-HCl, pH 8.0, 200 mM NaCl, 10 mM imidazole, 5 µg/mL DNase I, 2-3 tablets of Pierce™ Protease Inhibitor (ThermoFisher Scientific, 88266)) and lysed by passing the resuspended pellet through a French Press at 18,000 psi. Lysed cells were spun for 90 min at 12,000*xg*, 4°C. The resulting cell pellet from lysis was discarded, and the supernatant was filtered using a 0.45 µM filter membrane. Filtered supernatant was loaded by gravity onto two columns packed with 5 mL HisPur™ Ni-NTA Resin (ThermoFisher Scientific, 88221) and preequilibrated with 10 column volumes of low imidazole buffer (25 mM Tris-HCl, pH 8.0, 200 mM NaCl, 10 mM imidazole). Resulting flow through was collected, and the columns were washed with 10 column volumes of low imidazole buffer. His-PcsB-CC proteins were eluted as 5 mL fractions from the column using high imidazole buffer (25 mM Tris-HCl, pH 8.0, 200 mM NaCl, 400 mM imidazole). 40 µL of resulting fractions were mixed with an equal volume of 2X Laemelli buffer and run on an SDS-PAGE gel (5-14% gradient gel) at 150 V for 1 hour. The resulting gel was stained with Coomassie blue and observed for the presence of His-PcsB-CC (running at approximately 25 kDa). Fractions containing His-PcsB-CC were combined, and 1 mM DTT, 1 mM EDTA, and TEV protease were added. The mixture was then incubated at 4°C overnight with rotation. The next day, an SDS-PAGE gel was run to determine if the TEV protease cleaved the His tag off of PcsB-CC (PcsB-CC ran at approximately 24 kDa). PcsB-CC was then dialyzed into low imidazole buffer overnight and the Ni-NTA column was repeated the next day, collecting the flow-through as 5 mL fractions. The resulting fractions were again run on SDS-PAGE gel (5-14%

gradient gel) at 150 V for 1 hour. Fractions observed to contain PcsB-CC were concentrated to 2.5 mL using an Amicon Ultra-15 centrifugal filter unit with a 10 kDa cut-off (Millipore Sigma) and buffer exchanged by a Sephadex G-25 PD-10 desalting column (GE Healthcare) into Buffer A (25 mM Tris-HCl, pH 8.0, 50 mM NaCl). Sample was then loaded onto a 6 mL Resource Q column (GE Healthcare) on an ÄKTA pure chromatography system (GE Healthcare) pre-equilibrated with Buffer A. The column was run at 6 mL/min, and PcsB-CC was eluted using a 20 column volume gradient from 0-50% Buffer B (25 mM Tris-HCl, pH 8.0, 1 M NaCl), and step to 100% B over 5 column volumes. Resulting fractions were examined for presence of PcsB-CC by running 20 µL samples mixed with an equal volume of 2X Laemelli sample buffer on an SDS-PAGE gel (5-14% gradient gel) at 150 V for 1 hour. The resulting gel was stained with Coomassie blue and observed for the presence of PcsB-CC (running at approximately 24 kDa). Total PcsB-CC amount was determined by measuring the resulting sample's absorbance at 280 nm using a Shimadzu UV-2401 PC UV-Vis recording spectrophotometer, and calculated using Beer's Law and the extinction coefficient of PcsB-CC (2980 $M^{-1}cm^{-1}$) at 280 nm. The extinction coefficient was determined by the input of the amino acid sequence into the ExPASy ProtParam tool (http://web.expasy.org/protparam/). Purified proteins were stored at -70°C. Purified PcsB-CC (47-267) had a double band that co-migrated on an SDS-PAGE gel. MALDI and LC-MS/MS determined that full length PcsB-CC (47-267) was present in both of these bands, indicating that we had only full-length PcsB-CC (47-267) present in our purified samples.

Expression and purification of His tagged PcsB coiled coil domain *E. coli* strains containing plasmids expressing His-PcsB-CC (47-267) and His-PcsB-CC (47-254) (Table S1) were induced, grown, and purified in the same manner as outlined for N^{15} or unlabeled PcsB coiled coil domain, with the following exceptions. His-PcsB-CC (47-267) was purified using the same procedure, with the exception that no TEV cleavage, dialysis, or second gravity Ni-NTA column was performed. His-PcsB-CC (47-254) was also purified in the same way as His-PcsB-CC (47-267), with the exception that after the Resource Q column His-PcsB-CC(47-267) was buffer exchanged into $FtsX_{ECL1}$ NMR buffer, purified protein concentration was measured, and then stored at -70°C.

^{1}H-^{15}N TROSY (Transverse relaxation-optimized) NMR Spectroscopy ^{15}N PcsB-CC (47-267) and ^{15}N PcsB-CC (47-254) TROSY spectra were recorded at 298 K on a Varian (Agilent) DDR 800 MHz spectrometer equipped with a cryogenic probe in the METACyt Biomolecular NMR Laboratory at Indiana University Bloomington. NMR samples contained 50 mM potassium phosphate, pH 7.0, 50 mM NaCl, 10 % v/v D2O, and 50-100 μM ^{15}N PcsB-CC (47-267) or ^{15}N PcsB-CC (47-254). For experiments detecting $FtsX_{ECL1}$ binding, ^{15}N PcsB-CC(47-254) was kept at 50 μM concentration, and ^{1}H-^{15}N TROSY spectra were recorded with the following concentrations of $FtsX_{ECL1}$: 0, 50 μM, 200 μM, and 400 μM. nmrPipe and Sparky ver. 3.114 were used for data processing and analysis.

Isothermal Titration Calorimetry ITC titrations were performed using a MicroCal VP-ITC calorimeter (Malvern Panalytical) using 330 μM wild-type or 330 μM L115A/M119A $FtsX_{ECL1}$ in the syringe and 20 μM His-PcsB-CC(47-267) in the reaction cell. 6 μL injections were made at the default rate of 2 μL per second and 180 s was allowed for

equilibration. For all experiments, the buffer was 50 mM potassium phosphate, 50 mM NaCl, 0.5 mM EDTA, pH 7.0. All experiments were run at 25.0 °C. All reactions were conducted two times (including controls). The Origin 7.0 Software package (OriginLab Corp.) provided by MicroCal was used for fitting procedures, and a one state model was used for all fittings.

Circular Dichroism Experiments All circular dichroism (CD) spectra were recorded on a Jasco J-715 Circular Dichrosim Spectropolarimeter equipped with a Peltier temperature control accessory in the Physical Biochemistry Instrumentation Facility at Indiana University, Bloomington. $FtsX_{ECL1}$ CD samples or PcsB-CC CD samples at 5 to 10 μM dialyzed into 50 mM sodium phosphate, pH 7.0, 50 mM NaF. Spectra were recorded in a 1 mM cuvette, with the following parameters: sensitivity at 100 mdeg, starting and ending wavelength at 180-250 nm, data pitch at 1.0 nm, scanning mode continuous, scanning speed at 200 nm/min, response at 1 sec, band width at 1 nm, wavelength interval at 1 nm, and temperature at 25°C for PcsB-CC CD experiments and 15°C for $FtsX_{ECL1}$ CD experiments. Raw data output of spectra was baseline subtracted and plotted using GraphPad Prism 5 (GraphPad Software, Inc.).

Analysis of proteins by LC-MS ESI and LC-MS/MS To confirm protein identity and molecular weight, wild-type $FtsX_{ECL1}$, W123A, F126A, N131A $FtsX_{ECL1}$, L115A/M119A $FtsX_{ECL1}$,^{15}N PcsB-CC (47-254), ^{15}N PcsB-CC (47-267), His-PcsB-CC (47-267), and His-PcsB-CC (47-267) were purified as outlined previously in *Supplemental Materials and Methods*. 25-75 μM purified protein was run as a 5 μL injection on a Waters/Micromass LCT Classic KC-379 (Waters) equipped with a Zorbax C4-SB300 (0.5 x 150 mm) column (Phenomenex), with a 20 minute linear gradient of 90% solvent

A (95% H_2O, 5% CH_3CN, 0.1% HCOOH) to 90% solvent B (95% CH_3CN, 5% H_2O, 0.1% HCOOH). Resulting LC-MS data was analyzed using MassLynx (Waters). Expected molecular weights as calculated by the online ExPASy ProtParam tool (as previously referenced) and the addition of the appropriate isotope weight were compared to experimental molecular weights to confirm protein identify.

To confirm the identity of the two protein bands resulting from the isolation of PcsB-CC (47-267), 5 µg of pure protein sample was mixed with 20 µL of 2X Laemelli sample buffer, and boiled for 10 minutes. The resulting sample was run on 15% SDS-PAGE gel for 2 hrs at 150 V. The gel was incubated and stained with Coomassie Blue, and then examined for the presence of the two protein bands. Gel fragments for each band were extracted using a razor blade, by hand, and placed in a 1.5 mL Eppendorf tube. Gel fragments were given to the IU Biological Mass Spec Facilty, who extracted and ran samples by LC-MS/MS (http://www.chem.indiana.edu/bms/).

Multiple sequence alignment of FtsX(ECL1)*Spn* with the FtsX amino acid sequence from other organisms To obtain an amino acid multiple sequence alignment of FtsX(ECL1)$_{Spn}$ with FtsX from other organisms, FtsX amino acid sequences from *S. pneumoniae* D39, *M. tuberculosis* H37Rv, *E. coli* K12, *S. gordonii* Challis CH1, *S. mitis* SK637, *S. mutans* UA519, *S. pyogenes* MGAS1882, *B. cereus* B4264, and *B. subtilis* 2014-3557 were obtained from PubMed Gene Database on 9 July 2017. Amino acid sequences were entered into the Clustal Omega webserver (http://www.ebi.ac.uk/Tools/msa/clustalo/). The resulting multiple sequence alignment was entered into the ESPript 3.0 webserver (http://espript.ibcp.fr/ESPript/ESPript/) to

obtain an overlay of the known secondary structure from $FtsX_{Mtb}$ with the multiple sequence alignment.

Construction of *S. pneumoniae* strains. Transformation of D39 Δ*cps* (IU1945) to obtain derivative strains (Table S1) was performed with linear DNA amplicons made by fusion PCR as described previously (8). Primers used to construct D39 Δ*cps* derivative strains are listed in Table S1. Constructed strains were confirmed by PCR and DNA sequencing of the chromosomal region corresponding to the linear amplicon and surrounding regions.

Construction of FtsX depletion strain and strains conditionally expressing point mutants. To construct the FtsX merodiploid strain, we constructed a strain that linked the zinc-inducible promoter to *ftsX*$^+$ at the *bgaA* site and knocked out *ftsX* at the native locus by replacement with an antibiotic cassette (Δ*ftsX*::P-*aad9*) (Table S1). To construct strains conditionally expressing point mutants, a *rpsL1* strain (IU1824) with P_{Zn}-*ftsX*$^+$ at the *bgaA* site was transformed with [*kan-rpsL*$^+$]-*ftsX*$^+$ at the native *ftsX* site. This strain expressed both wild type *ftsX*$^+$ and [*kan-rpsL*$^+$]-*ftsX*$^+$ when zinc was added to the media. We then transformed markerless alleles of *ftsX* with point mutations in the presence of zinc, selecting for streptomycin resistance and kanamycin sensitivity. For strains and primers, see Table S1.

Western blotting analysis and immunodetection. For detection of FtsX expression levels, the following sample isolation procedure was used. Strains for western blot analysis were inoculated from frozen glycerol stocks into BHI, serial diluted, and incubated for 10-12 hours statically at 37°C in 5% CO2 overnight. The next day, cultures from $OD_{620} \approx 0.05$ to 0.4 were diluted into fresh BHI to $OD_{620} \approx 0.003$ in 25 mL

volumes, and two identical cultures for each strain were prepared, one with 0.45 mM $ZnCl_2$ and 0.045 $MnSO_4$ and one without. Growth was monitored turbidimetrically with a Genesys 2 spectrophotometer (Thermo Scientific), and at 6 hours post dilution cultures were spun at 12,000*xg* for 5 min at 4°C. Supernatants were discarded and pellets were resuspended in 1.5 mL of 20 mM potassium phosphate, pH 7.5, 140 mM NaCl. Samples were transferred to individual 2 mL Lysing Matrix B Fast Prep tubes (MP Biomedicals, 116911050) and samples were lysed at 4°C using a FastPrep-24 (MP Biomedicals) with the following settings repeated three times: Speed, 6.0 m/s, Adapter, 24x2, Time 40 sec. After the third run, samples were placed immediately on ice and spun at 12,000*xg* for 1 min at room temperature. Samples were then transferred to 3.2 mL Beckman polypropylene ultracentrifuge tubes (Beckman Coulter, 362333) and centrifuged in a TLA-100.4 rotor in an Optima TLX ultracentrifuge (Beckman Coulter) at 100,000*xg* for 45 min at 4°C. After ultracentrifugation, supernatants were discarded and pelleted membranes were resuspended in 100-400 µL membrane resuspension buffer (20 mM potassium phosphate, pH 7.5, 140 mM NaCl, 0.02% n-Dodecyl β-D-maltoside). The Bio-Rad DC™ protein assay kit I was used to determine total protein concentrations of samples using a standard curve of 0.1 to 3.0 mg/mL of BSA. Absorbance of samples (750 nm) was determined in a 96-well plate reader (Synergy H1 Hybrid Reader, BioTek), and protein concentration was determined. Samples were diluted with 2X Laemlli SDS loading buffer (Bio-Rad) and incubated at 95°C for 10 min. 12.5 µg of total protein was loaded per sample onto a 4-15% precast gradient SDS-PAGE gel (Bio-Rad, 4561084) and subjected to electrophoresis for 1 hour at 150 V. Proteins were transferred to a nitrocellulose membrane and probed with polyclonal rabbit α-FtsX

(1:500, see experimental procedures for production) primary antibody and ECL anti-rabbit IgG horseradish peroxidase linked whole antibody (1:10,000, GE Healthcare, NA93AV) secondary antibody in TBST (Tris-Buffered Saline, 0.02% Tween). Antibody signal was detected by incubating membranes with Amersham ECL Western Blotting Detection Reagent (GE Healthcare, RPN2106) and detecting chemiluminescent signals from protein bands using an IVIS imaging system with a 1 min exposure as described in previously (9).

To verify protein loading was similar across all lanes, after detection blots were stripped using 15 mL of Restore PLUS Western Blot Stripping Buffer (Thermo Fisher Scientific, 46430) for 15 minutes at room temperature, and rinsed two times with TBST briefly. Alternatively, blots were placed in 45 mL of Stripping Buffer X (2% SDS, 62.5 mM Tris-HCl, pH 6.8, 110 mM β-mercaptoethanol) for 45 min at 50°C. Blots were briefly rinsed with water twice, and washed with TBST for 5 min twice, at room temperature. After stripping, blots were then probed with α-MreC primary antibody as previously described (10) and ECL anti-rabbit IgG horseradish peroxidase linked whole antibody as stated previously. Antibody signal was detected as stated previously in *Supplemental Materials and Methods.*

Production of α-FtsX $FtsX_{ECL1}$ was purified as outlined previously in *Supplemental Materials and Methods*. For antibody production, purified $FtsX_{ECL1}$ was sent to ThermoFisher Scientific (https://www.thermofisher.com/us/en/home/life-science/antibodies/custom-antibodies/custom-antibody-production/custom-polyclonal-antibody-production.html). Polyclonal antibodies were produced in rabbits, using the 70 day protocol. Day 58 raw serum was used as ‘polyclonal rabbit α-FtsX’.

REFERENCES

1. Sheffield P, Garrard S, Derewenda Z. 1999. Overcoming expression and purification problems of RhoGDI using a family of "parallel" expression vectors. Protein Expr Purif 15:34-9.
2. Fu Y, Bruce KE, Rued B, Winkler ME, Giedroc DP. 2016. 1H, 13C, 15N resonance assignments of the extracellular loop 1 domain (ECL1) of *Streptococcus pneumoniae* D39 FtsX, an essential cell division protein. Biomol NMR Assign 10:89-92.
3. Guntert P. 2004. Automated NMR structure calculation with CYANA. Methods Mol Biol 278:353-78.
4. Shen Y, Delaglio F, Cornilescu G, Bax A. 2009. TALOS+: a hybrid method for predicting protein backbone torsion angles from NMR chemical shifts. J Biomol NMR 44:213-23.
5. Zweckstetter M. 2008. NMR: prediction of molecular alignment from structure using the PALES software. Nat Protoc 3:679-90.
6. Stamsas GA, Havarstein LS, Straume D. 2013. CHiC, a new tandem affinity tag for the protein purification toolbox. J Microbiol Methods 92:59-63.
7. Sanchez-Puelles JM, Sanz JM, Garcia JL, Garcia E. 1992. Immobilization and single-step purification of fusion proteins using DEAE-cellulose. Eur J Biochem 203:153-9.
8. Tsui HC, Mukherjee D, Ray VA, Sham LT, Feig AL, Winkler ME. 2010. Identification and characterization of noncoding small RNAs in *Streptococcus pneumoniae* serotype 2 strain D39. J Bacteriol 192:264-79.

9. Wayne KJ, Sham LT, Tsui HC, Gutu AD, Barendt SM, Keen SK, Winkler ME. 2010. Localization and cellular amounts of the WalRKJ (VicRKX) two-component regulatory system proteins in serotype 2 *Streptococcus pneumoniae*. J Bacteriol 192:4388-94.

10. Rued BE, Zheng JJ, Mura A, Tsui HT, Boersma MJ, Mazny JL, Corona F, Perez AJ, Fadda D, Doubravova L, Buriankova K, Branny P, Massidda O, Winkler ME. 2017. Suppression and synthetic-lethal genetic relationships of DeltagpsB mutations indicate that GpsB mediates protein phosphorylation and penicillin-binding protein interactions in *Streptococcus pneumoniae* D39. Mol Microbiol 103:931-957.

11. Sung CK, Li H, Claverys JP, Morrison DA. 2001. An rpsL cassette, janus, for gene replacement through negative selection in *Streptococcus pneumoniae*. Appl Environ Microbiol 67:5190-6.

12. Lanie JA, Ng WL, Kazmierczak KM, Andrzejewski TM, Davidsen TM, Wayne KJ, Tettelin H, Glass JI, Winkler ME. 2007. Genome sequence of Avery's virulent serotype 2 strain D39 of *Streptococcus pneumoniae* and comparison with that of unencapsulated laboratory strain R6. J Bacteriol 189:38-51.

13. Sham LT, Barendt SM, Kopecky KE, Winkler ME. 2011. Essential PcsB putative peptidoglycan hydrolase interacts with the essential $FtsX_{Spn}$ cell division protein in *Streptococcus pneumoniae* D39. Proc Natl Acad Sci U S A 108:E1061-9.

14. Tsui HC, Zheng JJ, Magallon AN, Ryan JD, Yunck R, Rued BE, Bernhardt TG, Winkler ME. 2016. Suppression of a deletion mutation in the gene encoding essential PBP2b reveals a new lytic transglycosylase involved in peripheral

peptidoglycan synthesis in *Streptococcus pneumoniae* D39. Mol Microbiol 100:1039-65.

APPENDIX FIGURE LEGENDS

Figure A1: Cells expressing FtsX(N131D) become shorter and rounder, while cells expressing FtsX(E109Q) have no phenotype. A) Amino acid changes made mapped to the structure of $FtsX_{ECL1}$. The α carbon of each residue is shown as a colored sphere. The orange to red coloring on the $FtsX_{ECL1}$ structure represents the peak height changes in the ^{1}H-^{15}N HSQC spectra upon addition of 2 molar equivalents of PcsB-CC to $FtsX_{ECL1}$. B) Representative growth curve of strains expressing FtsX(E109Q) and FtsX(N131D). These strains were grown with or without 0.45 mM ZnCl2 with 0.045 mM $MnSO_4$ (indicated as +Zn). Strains shown are as follows: black circle, D39 *rpsL1* Δ*cps* wild-type parent (1, IU1824); grey circle, IU1824 +Zn; dark blue square, D39 *rpsL1* Δ*cps* Δ*ftsX*::P-*aad9*//*bgaA*::*tet*-P_{Zn}-*ftsX*$^{+}$ (2, IU12376); light blue square, IU12376 +Zn; orange triangle, D39 *rpsL1* Δ*cps* *ftsX*(E109Q)// *bgaA*::*tet*-P_{Zn}-*ftsX*$^{+}$ (3, IU13088); light orange triangle, IU13088 +Zn; dark pink inverted triangle, D39 *rpsL1* Δ*cps* *ftsX*(N131D)//*bgaA*::*tet*-P_{Zn}-*ftsX*$^{+}$ (4, IU13089); pink inverted triangle, IU13089 +Zn. This growth curve was repeated three times with similar results. B) Representative images of strains at 6 hours growth. The genotype of the strain shown is indicated above each panel. No Zn or +Zn indicates if Zn/Mn was added. % indicates the percentage of cells in the population that are morphologically similar to the images shown. Greater than 50 cells per strain, condition, and experimental repeat were analyzed. These experiments were performed three times independently with similar results. Scale bar shown is 1 μM. D) Length, width, aspect ratio, and relative cell volume at 6 hours growth. Strains are indicated according to the numbering in panel B. Greater than 50 cells were measured per strain and condition over two experimental replicates. For statistical analysis, a

Kruskal-Wallis test (one-way ANOVA) with Dunn's multiple comparison post-test was used to determine if length, width, aspect ratio, and relative cell volume were significantly different between strains and conditions. ns=non significant, * = $p<0.05$, ** = $p<0.005$, *** = $p<0.0005$.

Figure A2: Cells expressing FtsX $(Gly_3Ser)_2$ insertion after 78 have mild morphological defects, and no growth defects. $(Gly_3Ser)_2$ represents an amino acid insertion consisting of the sequence GSSSGSSS. A) Site of amino acid insertion mapped to the structure of $FtsX_{ECL1}$. The α carbon of two residues (78 and 79) between which $(Gly_3Ser)^2$ was inserted is shown as a colored sphere. The orange to red coloring on the $FtsX_{ECL1}$ structure represents the peak height changes in the ^1H-^{15}N HSQC spectra upon addition of 2 molar equivalents of PcsB-CC to $FtsX_{ECL1}$. B) Representative growth curve of strains expressing $FtsX(Gly_3Ser)_2$ versus FtsX depletion and wild-type strains. These strains were grown with or without 0.45 mM $ZnCl_2$ supplemented with 0.045 mM $MnSO_4$ (indicated as +Zn). Strains shown are as follows: black circle, D39 *rpsL1* Δ*cps* wild-type parent (1, IU1824); grey circle, IU1824 +Zn; dark blue square, D39 *rpsL1* Δ*cps* Δ*ftsX*::P-*aad9*//*bgaA*::*tet*-P_{Zn}-*ftsX*$^+$ (2, IU12376); light blue square, IU12376 +Zn; dark green triangle, D39 *rpsL1* Δ*cps* *ftsX*$(Gly_3Ser)_2$//*bgaA*::*tet*-P_{Zn}-*ftsX*$^+$ (3, IU13463); light green triangle, IU13463 +Zn. This growth curve was repeated two times with similar results. C) Representative images of strains at 6 hours growth. The genotype of the strain shown is indicated above each panel. No Zn or +Zn indicates if Zn/Mn was added. % indicates the percentage of cells in the population that are morphologically similar to the images shown. Greater than 50 cells were counted per experimental run, per condition and strain. These experiments were performed two times independently with similar results.

Scale bar is equal to 1 μM. D) Length, width, aspect ratio, and relative cell volume of strains at 6 hours growth. Strains are indicated according to the numbering in panel B. Greater than 50 cells were measured per strain and condition over two experimental replicates. For statistical analysis, a Kruskal-Wallis test (one-way ANOVA) with Dunn's multiple comparison post-test was used to determine if length, width, aspect ratio, and relative cell volume were significantly different between strains and conditions. ns=non significant, * = p<0.05, ** = p<0.005, *** = p<0.0005.

Figure A3: Expression of FtsX with an amino acid insertion in $FtsX_{ECL1}$ causes morphological and growth defects similar to FtsX depletion. A) Representative growth curve of strains expressing $FtsX_{ECL1}$ with an insertion of sequence GGGSGGGS after residue 51 (denoted as N-term ECL1 $(Gly_3Ser)_2$). These strains were grown with or without 0.45 mM $ZnCl_2$ with 0.045 mM $MnSO_4$ (indicated as +Zn in the growth curve legend). Strains shown are as follows: black circle, D39 *rpsL1* Δ*cps* wild-type parent (1, IU1824); grey circle, IU1824 +Zn; dark blue square, D39 *rpsL1* Δ*cps* Δ*ftsX::*P-*aad9*//*bgaA*::*tet*-P_{Zn}-*ftsX*$^+$ (2, IU12376); light blue square, IU12376 +Zn; dark pink triangle, D39 *rpsL1* Δ*cps ftsX N-term ECL1* $(Gly_3Ser)_2$//*bgaA*::*tet*-P_{Zn}-*ftsX*$^+$ (3, IU12629); pink triangle, IU12629 +Zn. This growth curve was repeated three times with similar results. B) Representative growth curve of strains expressing $FtsX_{ECL1}$ with an insertion of GGGSGGGS after residue 173 (denoted as C-term ECL1 $(Gly_3Ser)_2$). Strains were grown as described in part A of this figure legend. Strains shown are as follows: black circle, D39 *rpsL1* Δ*cps* wild-type parent (1, IU1824); grey circle, IU1824 +Zn; dark blue square, D39 *rpsL1* Δ*cps* Δ*ftsX::*P-*aad9*//*bgaA*::*tet*-P_{Zn}-*ftsX*$^+$ (2, IU12376); light blue square, IU12376 +Zn; orange inverted triangle, D39 *rpsL1* Δ*cps ftsX C-term ECL1*

$(Gly_3Ser)_2$//*bgaA*::*tet*-P_{Zn}-*ftsX*$^+$ (4, IU12869); light orange inverted triangle, IU12869 +Zn. This growth curve was repeated three times with similar results. C) Representative images of strains at 6 hours growth. The genotype of the strain shown is indicated above each panel. No Zn indicates strains grown without Zn, and +Zn indicates strains growth with Zn. % indicates the percentage of cells in the population that are morphologically similar to the images shown. Greater than 50 cells were counted per experimental run, per condition and strain. These experiments were performed three times independently with similar results. Scale bar shown is equal to 1 μM. D) Length, width, aspect ratio, and relative cell volume of strains at 6 hours growth. Strains are indicated according to the numbering in panel A. Measurements for FtsX C-term ECL1 $(Gly_3Ser)_2$ are not shown, as this strain did not express FtsX in the absence of zinc. Greater than 50 cells were measured per strain and condition over two experimental replicates. For statistical analysis, a Kruskal-Wallis test (one-way ANOVA) with Dunn's multiple comparison post-test was used to determine if length, width, aspect ratio, and relative cell volume were significantly different between strains and conditions. ns=non significant, * = $p<0.05$, ** = $p<0.005$, *** = $p<0.0005$.

APPENDIX FIGURE A1

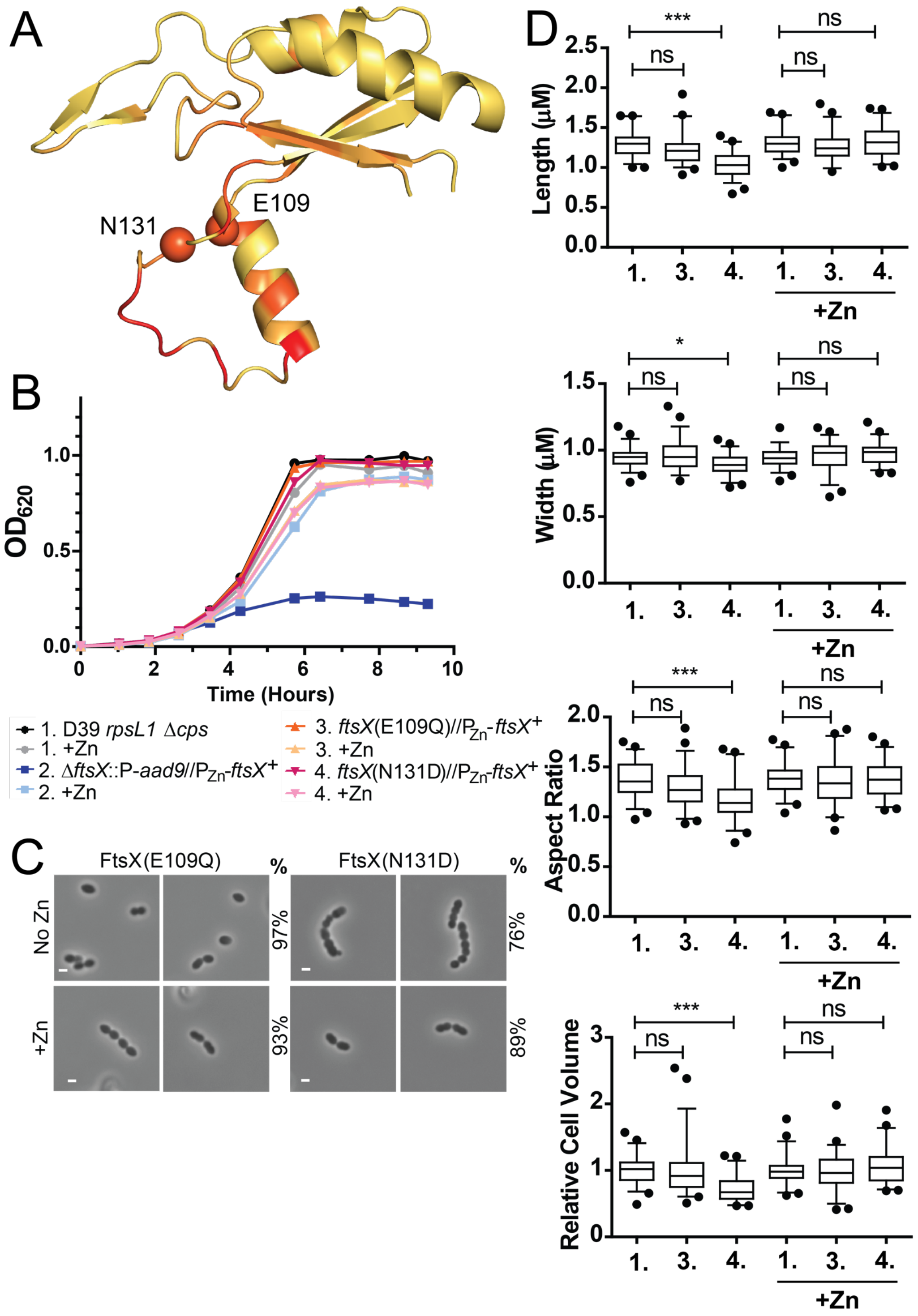

APPENDIX FIGURE A2

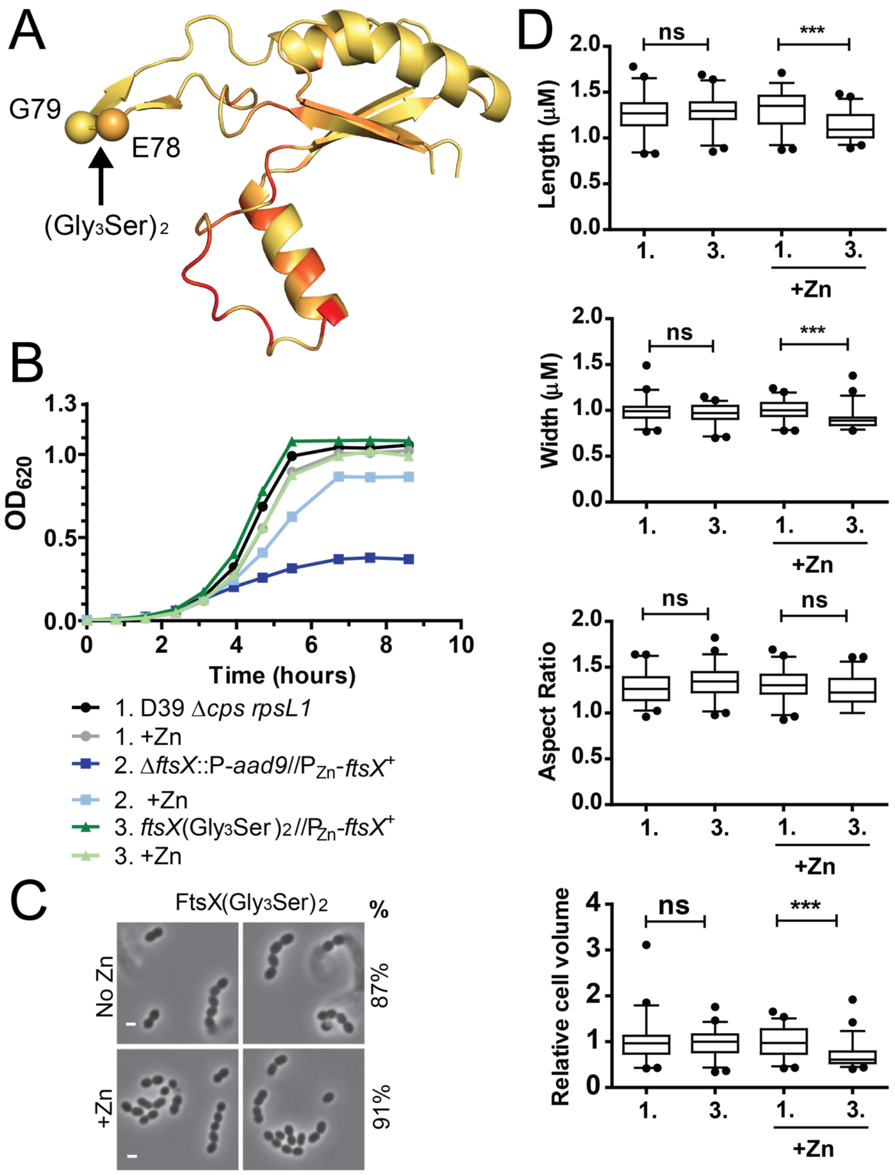

APPENDIX FIGURE A3

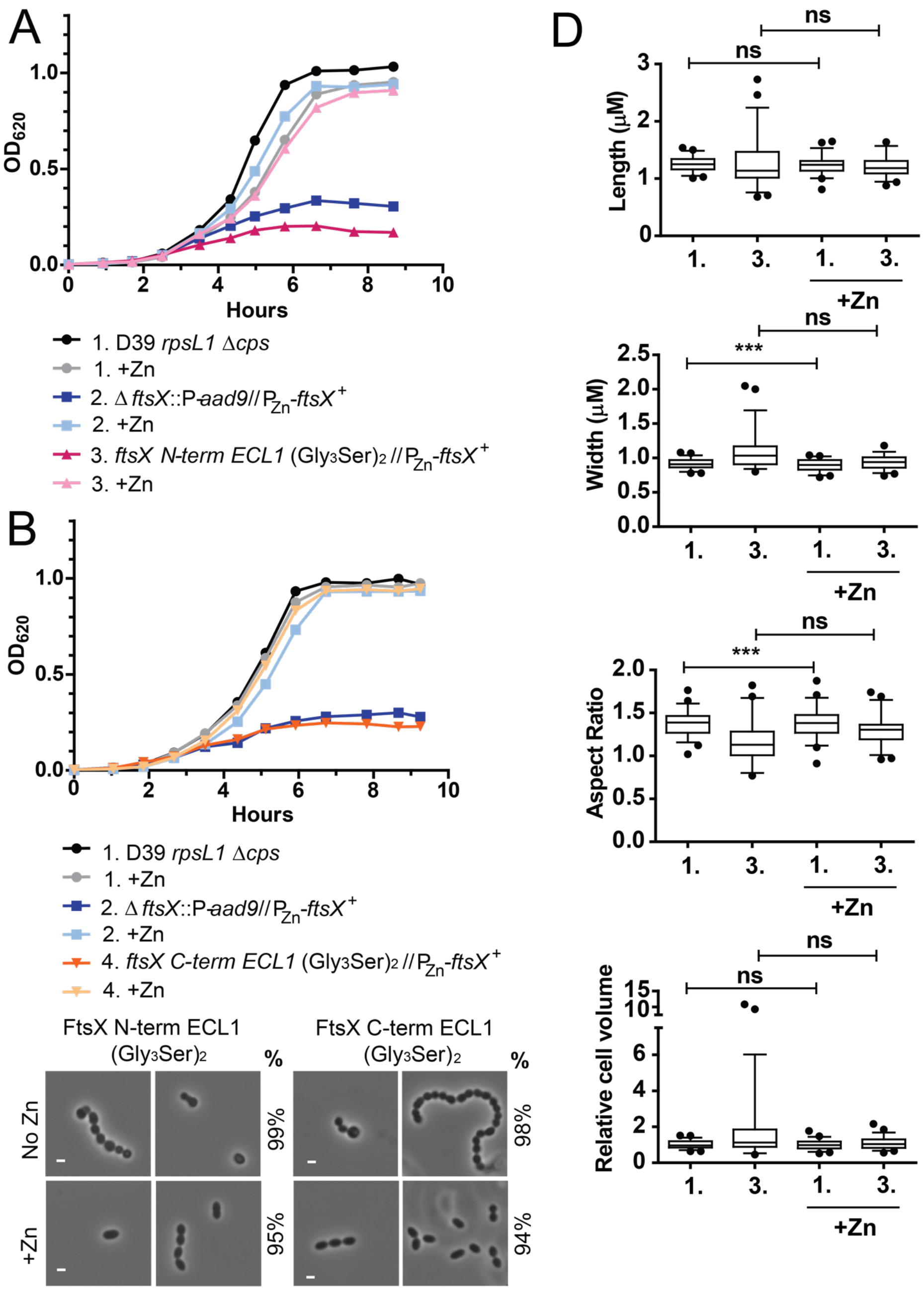

Supplement: TEXT S1 [file mBio.02622-18-s0001.pdf]
